# Antibacterial and Antifungal Alkaloids from Asian Angiosperms: Distribution, Mechanisms of Action, Structure-Activity, and Clinical Potentials

**DOI:** 10.3390/antibiotics11091146

**Published:** 2022-08-24

**Authors:** Mazdida Sulaiman, Khoshnur Jannat, Veeranoot Nissapatorn, Mohammed Rahmatullah, Alok K. Paul, Maria de Lourdes Pereira, Mogana Rajagopal, Monica Suleiman, Mark S. Butler, Mohammed Khaled Bin Break, Jean-Frédéric Weber, Polrat Wilairatana, Christophe Wiart

**Affiliations:** 1 Department of Chemistry, Faculty of Science, University of Malaya, Kuala Lumpur 50603, Malaysia; 2 Department of Biotechnology & Genetic Engineering, University of Development Alternative, Dhaka 1207, Bangladesh; 3 School of Allied Health Sciences and World Union for Herbal Drug Discovery (WUHeDD), Walailak University, Nakhon Si Thammarat 80160, Thailand; 4 School of Pharmacy and Pharmacology, University of Tasmania, Hobart, TAS 7001, Australia; 5 CICECO-Aveiro Institute of Materials & Department of Medical Sciences, University of Aveiro, 3810-193 Aveiro, Portugal; 6 Faculty of Pharmaceutical Sciences, UCSI University, Kuala Lumpur 56000, Malaysia; 7 Institute for Tropical Biology & Conservation, Universiti Malaysia Sabah, Kota Kinabalu 88400, Malaysia; 8 MSBChem Consulting, Brisbane, QLD 4005, Australia; 9 Department of Pharmaceutical Chemistry, College of Pharmacy, University of Hail, Hail 81411, Saudi Arabia; 10 UFR Sciences Pharmaceutiques, INRAE, Bordeaux INP, UR ŒNOLOGIE, EA 4577, USC 1366, ISVV, Université de Bordeaux, 210 Chemin de Leysotte, 33882 Villenave d’Ornon, France; 11 Department of Clinical Tropical Medicine, Faculty of Tropical Medicine, Mahidol University, Bangkok 10400, Thailand

**Keywords:** medicinal plants, antibacterial, antifungal, alkaloids, Asia

## Abstract

The emergence of multidrug-resistant bacteria and fungi requires the development of antibiotics and antifungal agents. This review identified natural products isolated from Asian angiosperms with antibacterial and/or antifungal activities and analyzed their distribution, molecular weights, solubility, and modes of action. All data in this review were compiled from Google Scholar, PubMed, Science Direct, Web of Science, ChemSpider, PubChem, and a library search from 1979 to 2022. One hundred and forty-one antibacterial and/or antifungal alkaloids were identified during this period, mainly from basal angiosperms. The most active alkaloids are mainly planar, amphiphilic, with a molecular mass between 200 and 400 g/mol, and a polar surface area of about 50 Å^2^, and target DNA and/or topoisomerase as well as the cytoplasmic membrane. 8-Acetylnorchelerythrine, cryptolepine, 8-hydroxydihydrochelerythrine, 6-methoxydihydrosanguinarine, 2′-nortiliacorinine, pendulamine A and B, rhetsisine, sampangine, tiliacorine, tryptanthrin, tylophorinine, vallesamine, and viroallosecurinine yielded MIC ≤ 1 µg/mL and are candidates for the development of lead molecules.

## 1. Introduction

The resistance of bacteria and fungi to antimicrobial agents necessitates the continuous development of antibiotics and antifungal agents with original chemical frameworks that may come from flowering plants. The flowering plants also termed angiosperms comprise 11 major taxa or clades grouped into three groups: (i) basal angiosperms including Protomagnoliids, Magnoliids, Monocots, Eudicots; (ii) core angiosperms including Core Eudicots, Rosids, Fabids, Malvids; and (iii) upper angiosperms including Asterids, Lamiids, and Campanulids [1]. Within each clade, plants yield both non-specific and specific secondary metabolites such as alkaloids to control the growth of phytopathogenic bacteria and fungi. These antimicrobial principles fall into two main groups: phytoanticipins and phytoalexin. Phytoanticipins are antimicrobial compounds in plants that are present before phytopathogenic microorganism challenge or inactive immediate precursors stored in healthy tissues that are converted into antimicrobial metabolites known as phytoalexins [2]. Phytoanticipins and phytoalexins containing primary, secondary, tertiary, or quaternary amines are called alkaloids, which belong to various chemical classes, principally amides, indoles, piperidines, quinolines, isoquinolines, pyrrolidines, imidazoles, diterpenes, sesquiterpeness, and steroidal alkaloids [3].

Phytopathogenic Gram-negative bacteria are more resistant to alkaloids than Gram-positive bacteria due, at least in part, to an outer hydrophilic and negatively charged layer of lipopolysaccharides [4]. In Gram-negative bacteria, porins allow for the entry of water and nutrients through the outer layer and hydrophilic and amphiphilic xenobiotics with a molecular mass below 600 g/mol xenobiotics without nutritional or physiological benefit or toxins are actively cleared from the cytoplasm by efflux pumps [5]. The assessment of the antibacterial and antifungal strength of isolated secondary metabolites in vitro is qualitatively appreciated by the measurement of the diameter of an inhibition zone and quantitively based on the minimum inhibiting concentration (MIC), and several thresholds of activity have been proposed [6,7,8,9]. Angiosperms produce alkaloids that inhibit the growth of both Gram-positive, Gram-negative bacteria, yeasts, and filamentous fungi and these principles are of potential therapeutic value. Among the factors that influence the antibacterial or antibacterial strength, and the targets of alkaloids are the molecular mass and water solubility [10].

Over the last 80 years, enormous research efforts have been devoted to the aim of identifying antibiotic or antifungal lead molecules in flowering plants globally, but to date, none of these have been developed as drugs. The present work attempts to provide a comprehensive review of the main findings regarding the antibacterial and antifungal alkaloids from Asian angiosperms. All data in this review were compiled from Google Scholar, PubMed, Science Direct, Web of Science, ChemSpider, PubChem, and a library search from 1979 to 2022 and were analyzed to address the following points: (i) distribution; (ii) strongest principles identified; (iii) spectrum of activity; (iv) influence of molecular mass, water solubility, and polar surface; (v) the mode of action; (vi) structure–activity; and (vii) efflux pump inhibition.

## 2. Distribution

**Figure 1:**
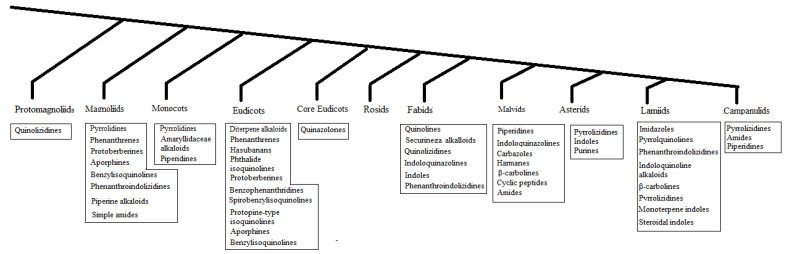
The phylogenetic distribution of antibacterial and/or antifungal alkaloids in angiosperms.

Regarding the distribution of antibacterial and antifungal alkaloids from Asian angiosperms (Figure 1), the following could be observed:

- All clades yielded antibacterial and/or antifungal alkaloids, except for the Rosids.
- Most antibacterial and/or antifungal alkaloids can be found in basal angiosperms, which are isoquinolines.
- Some clades yielded a specific class of antibacterial and/or antifungal alkaloids such as the Amaryllidaceae alkaloids in the Monocots, phenanthrene alkaloid in the Magnoliids, Securinega alkaloids in Fabids, carbazoles in the Malvids, and monoterpene indole alkaloids in the Lamiids.
- Core angiosperms and upper angiosperms use various classes of alkaloids and phytoalexins.
- Most antibacterial and/or antifungal alkaloids have been isolated from medicinal plants (Table 1, Table S1).
- Tripathi et al. (1994) observed changes in the antibacterial alkaloid concentrations in plants over time [11].

## 3. The Strongest Antibacterial and/or Antifungal Alkaloids Identified and Spectrum of Activity

Rios and Recio defined a crude extract with MIC greater than 1000 µg/mL as inactive and suggested interesting antibacterial activity for MICs of 100 µg/mL or lower [6]. Previously, Fabry et al. (1998) defined crude active extracts as having MIC values below 8000 µg/mL [7], while more recently, Kuete (2010) defined crude extracts with MIC values less than 100 µg/mL as active and MICs above 625 µg/mL as weakly active [9]. Here, a compound was very strongly antibacterial or antifungal for a MIC value below or equal to 1 µg/mL; strongly antibacterial (or antifungal for a MIC value above 1 and below or equal to 50 µg/mL; moderately antibacterial or antifungal for a MIC from 50 and below 100 µg/mL; weakly antibacterial (or antifungal) for a MIC from 100 and below 500 µg/mL; very weakly antibacterial or antifungal for a MIC ranging from 500 to below 2500 µg/mL; and inactive for a MIC value above 2500 µg/mL. Following this classification, 8-acetylnorchelerythrine, cryptolepine, 8-hydroxydihydrochelerythrine, 6-methoxydihydrosanguinarine, 2′-nortiliacorinine, pendulamine A and B, rhetsisine, sampangine, tiliacorine, tryptanthrin, tylophorinine, vallesamine, and viroallosecurinine showed very strong activities (Table S2). 

Looking at the spectrum of activity, most alkaloids showed activity against Gram-positive bacteria, followed by Gram-negative bacteria, yeasts, filamentous fungi, and mycobacteria (Table S2).

## 4. Influence of Molecular Mass 

The molecular mass of natural products dictates their ability to fit in the catalytic pockets of enzymes and to cross biological membranes. Here, low molecular mass molecules were defined with a molecular mass below 200 g/mol; medium molecular mass molecules were defined with a molecular mass from 200 to 400 g/mol; and high molecular mass molecules were defined for a molecular mass above 400 g/mol. Following this classification, we noted that principles with very strong activity against bacteria and fungi had a molecular mass mainly from 200 to 400 g/mol whereas very strong repressors of mycobacteria mainly had a mass above 400 g/mol (Table S2).

## 5. Influence of Solubility and Polar Surface

Because solubility is a fundamental criterion in which to consider the efficacy of alkaloids, there is need to use mathematical values. Log P is equal to the ratio of concentrations of a compound between octanol and water. Hydrophilic compounds (hydrophilic) have low or negative values (about −3) (compounds are mainly found in the water phase). Mid-hydrophilic compounds have a Log P close to 0 (the compound is equally partitioned between the octanol and water layers). Non-hydrophilic (hydrophobic, liposoluble) compounds have a high Log P (up to about 7) (note that lipophilic alkaloids tend to remain in and destabilize the cytoplasmic membrane of bacteria and fungi). However, Log P is only relevant for non-ionizable principles and for an ionized substance, a Log D is preferable (in terms of ADME), but the pH must be fixed. In some databases, one can find a Log P of 3 for the ionic alkaloid berberine, suggesting a lipophilic substance, which is not sensible. Since compounds destined for pharmaceutical development will mainly be exposed to physiological pH and often a weak base or a weak acid, we defined, at pH 7.4, lipophilic compounds for a negative Log D value of 4, amphiphilic (mid-polar) compounds for a Log D up to about 4.5, and lipophilic for a Log D above 4.5. Note that the Log D values given here are predicted values. Following this classification, we noted that principles with very strong activity against bacteria and fungi were mainly amphiphilic, whereas very strong antimycobacterial agents were mainly lipophilic (Table S2). Most antibiotics with very strong antibacterial and/or antifungal effects have a polar surface area around 50 Å (Table S2).

## 6. Mechanisms of Action and Structure-Activity Relationships

Most antibacterial and/or antifungal alkaloids from Asian angiosperms either bear a quinoline or an indole framework and the main mechanisms of action involve the targeting of the DNA, topoisomerases, and cytoplasmic membrane.

### 6.1. Alkaloids Targeting DNA and/or Topoisomerase

Some alkaloids including aristolochic acids bind to DNA or pyrrolizidine alkaloids, which form DNA cross-linking and DNA-protein cross-linking, leading to mutations [12,13]. Planar alkaloids tend to intercalate with bacterial DNA such as sanguinarine DNA [14], canthin-6-one [15], carbazoles [16], and tryptanthrin [17]. Cryptolepine intercalates into DNA and stimulates DNA cleavage by II [18] and was bacteriolytic for *Staphylococcus aureus* (NCTC 10788) [19]. Evodiamine inhibits topoisomerase I by stabilizing the enzyme-DNA covalent complex [20].

The mode of antibacterial and antifungal action of quinoline alkaloids mainly evokes interaction with DNA. Dictamnine binds to DNA under UV light [21]. Camptothecin stabilizes the topoisomerase I–DNA complex [22]. Liriodenine blocks topoisomerase II [23]. Berberine was active against *Actinobacillus pleuropneumoniae* and *Streptococcus agalactiae* (CVCC 1886) via DNA synthesis inhibition and the blockage of synthesis [24,25]. Anonaine induces DNA damage [26] as well as magnoflorine [27]. Aporphine alkaloids are planar and intercalate DNA and inhibit topoisomerase [28] as well as Amaryllidaceae alkaloids [29]. The quaternary ammonium ion of protoberberine alkaloids and their heterocyclic planar framework account for topoisomerase I inhibition [30].

During bacterial division, topoisomerase IV catalyzes the relaxation of the DNA chain and 6,6′-dihydroxythiobinupharidine blocks this enzyme in *S. aureus* [31]. Phenanthroindolizidine alkaloids interact with DNA [32]. In Gram-positive bacteria, topoisomerase IV is a target for bactericidal quinolone antibiotics [33]. Asian angiosperms produce a vast array of quinoline, isoquinoline, piperidine, and quinolizidine alkaloids, representing a fascinating reservoir of topoisomerase IV inhibitors. 

In summary, heterocyclic alkaloids with low to medium molecular mass, close to planar or planar, with the presence of a few hydroxy or ketone groups, almost always target DNA and/or RNA in bacteria and fungi. From this perspective, quinazolone alkaloids in the family Hydrangeaceae (order Saxifragales; clade Core Eudicots) could be examined for their antibacterial and or antifungal properties.

### 6.2. Alkaloids Targeting the Cytoplasmic Membrane

Phenanthroindolizidine alkaloids disturb the cytoplasmic membrane integrity [34]. The long alkyl chain of piperine and piperlongumine could penetrate the membrane of bacteria and fungi. In *Candida albicans*, piperine affects the membrane integrity, leading to oxidative stress followed by cell cycle arrest and apoptosis [35]. Marques et al. (2010) presented evidence that amides were more active against *Cladosporium cladosporoides* when the non-substituted aromatic ring, single double bonds, and substitution of nitrogen with alkyl groups were present [36]. In fungi, berberine targets the mitochondrial membrane [24]. Liriodenine in *Paracoccidioides brasiliensis* evoked cytoplasmic alterations and damage to the cell wall [37] while berberine evoked cytoplasmic insults in *Streptococcus agalactiae* (CVCC 1886) [34] and targeted the mitochondrial membrane of fungi [25].

### 6.3. Miscellaneous Targets

Aristolochic acids inhibited the H^+^-ATPase-mediated proton pump in *E. coli* [12]. Securinine induces mitotic block in cancer cells by binding to tubulin and inhibits microtubule assembly [38], therefore, microtubules could be involved in the antibacterial and/or antifungal properties of cytotoxic monoterpenoid indole alkaloids.

## 7. Efflux Pumps Inhibitors

*P*. *aeruginosa* and Gram-negative bacteria can resist a broad spectrum of natural products because they have extra classes of efflux pumps such as ABC (ATP binding cassette), RND (resistance nodulation cell-division), MF (major facilitator), SMR (small multidrug resistance) and MATE (multidrug and toxic compound extrusion) pumps [39]. ABC efflux pumps located in the cytoplasmic membrane of both Gram-positive and Gram-negative bacteria that use the energy derived from ATP hydrolysis to expel xenobiotics. RND efflux pumps located in the cytoplasmic and outer membrane of the Gram-negative bacteria (specific to Gram-negative bacteria) that expel xenobiotics using the H^+^ gradient (antiporters). MF efflux pumps located in the cytoplasmic membrane of both Gram-positive and Gram-negative bacteria that expel xenobiotics using the H^+^ gradient (antiporters). SMR efflux pumps located in the cytoplasmic membrane of both Gram-positive and Gram-negative bacteria that expel xenobiotics using the H^+^ gradient (antiporters). MATE efflux pumps located in the cytoplasmic membrane and are antiporters (the exit of the xenobiotic coincides with the entry of a Na^+^).

Tryptanthrin inhibits efflux P-glycoprotein in Caco-2 cells [16] and as such may inhibit bacteria and or fungal efflux pumps. Apocynaceous monoterpene indole alkaloids are often vasorelaxant [40], and therefore, with some inhibition levels of bacterial and or fungal efflux-pumps. For instance, the reserpine from *Rauvolfia serpentina* (L.) Benth. ex Kurz decreased the resistance of *S. aureus *(1199B, NorA hyperproducer) to ciprofloxacin and norfloxacin [10]. Reserpine is a calcium channel antagonist and an inhibitor of efflux pumps in Gram-negative bacteria and mycobateria [41]. From *Rauvolfia serpentina* (L.) Benth. ex Kurz, ajmaline and yohimbine are neuroactive and efflux pump inhibitors in Gram-negative bacteria [42]. Verapamil is another example of a calcium channel antagonist that inhibits the efflux pump in bacteria [42]. The reason why calcium channel antagonists have the tendency to inhibit the bacterial efflux pump could be because of the correlations between the bacterial efflux pumps and bacterial calcium transport [43]. Tetrandrine, which is a calcium channel antagonist in mammalian cells, inhibited the efflux pumps in *S*. *aureus* Rv2459 (jefA), Rv3728, and Rv3065 (mmr) efflux pumps in *Mycobacterium* species [44]. Therefore, natural products known for being calcium channel inhibitors should be screened as antibiotic potentiators. 4′-*O*-Methyldopamine inhibits NorA [45], showing that *N*-caffeoylphenalkylamide with the strongest efflux pump inhibitor activities presented hydroxyl substitution on the aromatic rings of the caffeic acid part and methoxy substitution on the aromatic ring of the dopamine moiety, which led to an increase in activity. Dopamine is a neurotransmitter, and it could be argued that neuroactive principles are a first line candidate for the development of efflux pump inhibitors. L-dopa increased the resistance of *C*. *neoformans* toward amphotericin B [46]. In line, erythrinan-type alkaloids and amide alkaloids in the family Piperaceae interact with GABAergic receptors and as such may be able to inhibit bacterial efflux pumps as in pellitorine, which at 16 µg/mL increased the sensitivity of *S. aureus* (RN4220) to erythromycin at 16 µg/mL via inhibition of the efflux pumps [5]. Canthin-6-one has a chemical structure with some similarity with serotonin and thus might be able to inhibit bacteria and/or fungal efflux pumps. In mammalian cells, tryptanthrin inhibits the expression of P-glycoprotein efflux pumps [46] and one could investigate its effect on the expression of efflux pumps in bacteria and fungi.

## 8. Amide Alkaloids

### 8.1. Simple Amide Alkaloids

Plants in the order Piperales (clade Magnoliids) yield antibacterial amide alkaloids with strong activity against Gram-positive bacteria (Figure 1). Pellitorine inhibited the growth of *Bacillus sphaericus* (ATCC 14577), *Bacillus subtilis* (ATCC 6051), *Staphylococcus aureus* (ATCC 9144), *Escherichia coli* (ATCC 25922), *Pseudomonas syringae* (ATCC 13457), and *S. typhimurium* (ATCC 23564) with the MIC values of 25, 12.5, 20, 150, 75, and 200 µg/mL, respectively [47]. Pellitorine (50 µg/disk) inhibited the growth of *Listeria monocytogenes* with an inhibition zone diameter of 9 mm and the MIC value of 500 µg/mL [48]. Pellitorine (20 µL of a 2 µg/mL solution/6 mm well) inhibited the growth of *Aspergillus flavus*, *Aspergillus fumigatus*, *Coniophora puteana*, *Fibrophoria vaillentii*, *Fusarium proliferatum*, and *Rhisopus* sp. with the inhibition zone diameters of 27, 29, 26, 28, 29, and 27 mm, respectively [49]. Piperlonguminine suppressed *B. sphaericus* (ATCC 14577), *B. subtilis* (ATCC 6051), *S. aureus* (ATCC 9144), *E. coli* (ATCC 25922), *P. syringae* (ATCC 13457), and *S. typhimurium* (ATCC 23564) with the MIC values of 20, 9, 12.5, 150, 75, and 175 µg/mL, respectively [47]. Piperlonguminine inhibited *Mycobacterium tuberculosis* with a MIC value of 50 µg/mL [50]. 8Z-*N*-isobutyleicosatrienamide and pellitorine had moderate potencies with *S. aureus* (MIC: 34 µM) [51]. Pellitorine restrained *M. tuberculosis* (H_37_Ra) with the MIC value of 25 µg/mL [52]. di-*p*-Coumaroyl-caffeoylspermidine weakly inhibited the mycelial growth of *Pyrenophora avenae* and *Blumeria graminis* [53]. In the clade Clampanulids, *Spilanthes paniculata* Wall. ex DC yielded N-Isobutyl-2 (E), 6 (Z), 8 (E)-decatrienamide (also known as spilanthol), which was bactericidal for *Streptococcus mutans* with MIC/MBC values of 125/125 µg/mL and weakly repressed *C. albicans* (ATCC 10231) [44]. The condensation of ferulic acid and dopamine yielded *N*-trans-feruloyl-4-methyldopamine 200 µg/disk that developed halos with a broad-spectrum of bacteria [54] and increased the susceptibility of multidrug-resistant *S. aureus* (overexpressing the multidrug efflux transporter NorA) to norfloxacin at 100 μg/mL [44]. 

### 8.2. Cyclopeptides

Plants in the Fabids and Malvids produce broad-spectrum antibacterial cyclic peptides such as frangulanine, which inhibited the growth *of S. aureus*, *B. subtilis*, *E. faecium*, *E. coli*, *E. cloacae*, *S. typhimurium*, and *P. aeruginosa* with the MIC values of 50, 50, 25, 6.2, 50, 50, 0.7, and 25 µg/mL, respectively [55]. Frangulanine inhibited the growth of *C*. *albicans* and *Saccharomyces cerevisae* with the minimum amounts of 25 and 50 µM, respectively [56]. Nummularine H isolated from the roots of *Ziziphus mauritiana* Lam. (family Celastraceae; order Celastrales; clade Fabids) inhibited the growth of *M*. *tuberculosis* (H_37_Ra) with the MIC of 4.5 μM [57].

## 9. Indole Alkaloids

### 9.1. Simple Indoles

#### 9.1.1. Brassicaceous Indoles

Plants in the family Brassicaceae yield antifungal sulfurated indole alkaloids such as caulilexin A from *Brassica oleracea* L. and at the concentration of 5 × 10^−4^ M, inhibited the growth of *Leptosphaeria maculans*, *Sclerotinia sclerotiorum*, and *Rhizoctonia solani* by 55, 100, and 100%, respectively [58]. The thiazoyl-substituted indole camalexin was active against *Alternaria brassicae* (MIC: 80 µg/mL) [59].

#### 9.1.2. β-Carbolines

The condensation of tryptophan and oxaloacetaldehyde yields strong broad-spectrum antibacterial and antimycobacterial β-carboline alkaloids in the clades Malvids and Lamiids. Canthin-6-one restrained the growth of *S*. *aureus* (1199B), *S*. *aureus* (XU212), and *S*. *aureus* (ENRSA-15) with the MIC values of 8, 8, and 32 µg/mL, respectively [60] as well as *Mycobacterium fortuitum* (ATCC 6841), *Mycobacterium smegmatis* (ATCC 14468), *M*. *smegmatis* (mc^2^22700), *Mycobacterium phlei* (ATCC 111758), and *Mycobacterium abcessus* (ATCC 19977) with the MIC values of 16, 8, 8, 8, and 16 µg/mL, respectively [60]. Rhetsinine very strongly suppressed *Xanthomonas oryxae* pv *oryzae*, *Xanthomonas oryxae* pv *oryzicola* with the EC_50_ values of 1 and 4.5 µg/mL, respectively [61]. The condensation of 2 *β*-carboline yielded borrerine yields in *Borreria verticillata* (L.) G. Mey. (clade Lamiids), which at the concentration of 50 µg/mL inhibited the growth of *S. aureus* and at 6 µg/mL restrained *Vibrio cholerae* [62]. 

#### 9.1.3. Carbazoles

The condensation of anthranilic acid and malonyl-CoA yields quinolinones that after prenylation and cyclization yield carbazole alkaloid phytoalexins in the family Nitrariaceae and Rutaceae in the order Sapindales (clade Malvids) (Figure 1). One such carbazole is glycozolidol (also known as 6-hydroxy-2-methoxy-3-methylcarbazole; 200 µg/mL/well), which restrained *S. aureus*, *Bacillus firmis*, *S. lutea*, *Agrobacterium tumefaciens*, and *Proteus vulgaris* [63]. 3-Formylcarbazole moderately inhibited the growth of *Mycobacterium* sp. [64]. Clausamine A (Log D = 3.9 at pH 7.4; molecular mass = 293.3 g/mol) inhibited the growth of MRSA (SK1), *S. aureus* (TISTR 1466), *E. coli* (TISTR 780), and *S. typhimurium* (TISTR 292) with the MIC values of 8, 32, 128, and 128 µg/mL, respectively [65]. Clausamine B very strongly inhibited the growth of MRSA (SK1) (MIC: 0.2 µg/mL) [65]. Clauszoline N inhibited the growth of MRSA (SK1), *E. coli* (TISTR 780), and *S. typhimurium* (TISTR 292) with the MIC values of 16, 64, and 128 µg/mL, respectively [65]. The prenylated carbazole clausine F very strongly inhibited MRSA (SK1) and *S. aureus* (TISTR 1466) with the MIC values of 4 µg/mL, respectively, and was moderately active for *E. coli* (TISTR 780) and *S. typhimurium* (TISTR 292) [65]. In the Rutaceae, lansine yielded a MIC of 14.3 µg/mL against *M. tuberculosis* (H37R*v*) [66]. Murrayamine J restrained *S. aureus* (ATCC 29213), *Bacillus cereus* (IIIM 25) with the IC_50_ values of 11.7 and 23.2 µM [67]. The prenylated carbazole girinimbine was active against *B. cereus* (IIIM 25) with the IC_50_ value of 3.4 µM as well as *S. aureus* and *Aspergillus niger* with the MIC values of 3.1 and µg/mL, respectively [68]. Koenimbine repressed *S. aureus* (ATCC 29213) and *B. cereus* (IIIM 25) with IC_50_ values of 17 and 22.5 µM [67] and koenigine was active against a variety of *Candida* sp. (MIC_90_: 12.5–100 μg/mL) [68]. 3-Formyl-1-1methoxycarbazole moderately repressed *S. aureus*, *B. subtilis*, *E. coli*, *P. vulgaris*, *A. niger*, and *C*. [68]. 3,3′-[Oxybis(methylene)]bis(9-methoxy-9*H*-carbazole) inhibited the growth of *Proteus vulgaris* and *C. albicans* with the MIC values of 6.2 and 25 µg/mL, respectively [69].In the Nitrariaceae, harmane is very strongly active against *Vibrio anguillarum* (MIC: 3.1 µg/mL), [70]. In a subsequent study, harman weakly inhibited the growth of *A. niger*, *C. albicans* (Neenah, 2010), *Cryptococcus gattii*, and *Cryptococcus neoformans* [71].

#### 9.1.4. Monoterpene Indole Alkaloids

Plants in the order Gentianales produce monoterpene indole alkaloids with moderate broad-spectrum antibacterial properties (Figure 1).

*Strictosidine-typeindole alkaloid glycosides*: The condensation of tryptophan and iridoid glycoside secologanin yields strictosidine, the precursor of 5α-carboxystrictosidine (Log D-3.1 at pH 7.4; molecular mass = 574.5 g/mol), which inhibited the growth of *M. tuberculosis *(H37Rv) with the MIC value of 26.3 µg/mL [72].

*Iboga indole alkaloids*: Iboga indole alkaloids are derived from strictosidine. Voacangine and ibogaine moderately inhibited the growth of *M. tuberculosis* and *Mycobacterium kansasi* [73]. 5-Oxocoronaridine and coronaridine were moderately active against repressed *E. coli*, *B. subtilis*, *A. flavus*, *A. niger*, and *Rhizoctonia phaseoli* [74]. The growth *E. coli*, *K. pneumoniae*, *S. aureus*, *S. pneumoniae * [74], *A. flavus*, *C. albicans*, and *R. phaseoli* was moderately inhibited by ibogamine [74]. *Penicillium chrysogenum* was moderately inhibited by 5-oxocoronaridine, 3-oxocoronaridine, and tabernamontamine [74]. 

*Vobasine-type indole alkaloids:* The growth of *A. niger* and *A. flavus* was moderately suppressed by vobasine [74]. Voacamine repressed *R. phaseoli*, *P. chrysogenum*, and *C. albicans* [74]. The growth was moderately hampered by vobasine [74].

*Isomalindan-type indole alkaloids*: Cadambine from *Neolamarckia cadamba *(Roxb.) Bosser (clade Lamiids) was weakly active toward *Staphylococcus epidermidis*, *S. aureus*, *B. cereus*, and *B. subtilis*, and *C. albicans* [75].

*Corynanthe indole alkaloids*: Strictosidine is the precursor to corynanthe indole alkaloids such as alstoniascholarine A, which hindered *K. pneumoniae*, *Providencia smaitii*, and *E. coli* with the MIC values of 12.5, 25, and 50 µg/mL, respectively [76] while alstoniascholarine E yielded the MIC values of 25, 25, and 25 µg/mL, against *K. pneumoniae*, *P. smaitii*, and *E. coli* with the MIC values of 100, 50, 50, respectively. Alstoniascholarine A and E moderately restrained *M. gypseum*, *E. floccosum*, and *T. mentagrophytes* [76]. Alstoniascholarine J repressed *E. floccosum* with the MIC of 31.2 µg/mL, and yielded the MICs of 3.1, 12.5, 12.5, 1.5, and 25 µg/mL with *P. aeruginosa*, *E. faecalis*, *K. pneumoniae*, *P. smaitii*, and *E. coli*, respectively [76]. Vallesamine and 5-hydroxy-19,20-*Z*-alschomine very strongly inhibited the growth of *E. faecalis* (ATCC 10541) and *P. aeruginosa* (ATCC 27853) with the MIC values of 1.5 and 0.7 µg/mL, respectively [77,78]. 

*Strychnos-type indole alkaloids*: Strictosidine is the precursor of Strychnos-type indole alkaloids such as tubotaiwine that moderately inhibited the growth of *M. tuberculosis* H37Rv (MIC: 100 µg/mL) [79].

#### 9.1.5. Miscellaneous

The condensation of anthranilic acid with isatin yielded the indoloquinazoline alkaloid tryptanthrin, which very strongly inhibited MRSA (MIC: 0.5 μg/mL) and *Malassezia furfur* (MIC: 4 μg/mL) [34,80]. Tryptanthrin is a broad-spectrum antifungal alkaloid that yielded the MIC values of 3.1, 3.1, 3.1, 3.1, 6.3, and 3.1 µg/mL, against *T. mentagrophytes*, *Trichophyton* *rubrum*, *Trichophyton* *tonsurans*, *Microsporum canis*, *M. gypseum*, and *E. floccosum* respectively [81,82]. Tryptanthrin was strongly fungistatic with *C. neoformans*, and *Cryptococcus deuterogattii* (MIC/MFC: 2/>64 and 8/32 μg/mL) [83].

Plants in the genus *Euodia* from tryptophan via the condensation of dihydronorharman and *N*-methylanthranilic acid yield evodiamine with weak potencies against *K. pneumoniae* (MDR, clinical strain) [84]. Dehydroevodiamine (molecular mass = 301.3 g/mol) from the fruits of *Euodia rutaecarpa * Benth very strongly restrained *X. oryxae* pv *oryzae* (EC_50_: 1.4 µg/mL) [61] 

In the family Apocynaceae (clade Lamiids), the condensation of anthranilic acid with indoxyl yielded the indoloquinoline alkaloid cryptolepine, which repressed *S. aureus* (NCTC 10788), *S. dysenteriae* (ATCC 13313), *V. cholerae* (ATCC 11623), *S. sclerotiorum*, and *Botrytis cinerea* with the MIC values of 5, 6.2, <1.5, 5.5, and 0.05 µg/mL, respectively [7,18,85,86]. 

## 10. Piperidine Alkaloids

### 10.1. Piperine Alkaloids

Plants in the family Piperaceae (clade Magnoliids) convert L-lysine into piperidine, which combines with piperoyl-CoA to yield the strong broad-spectrum antibacterial piperine alkaloids (Figure 1). Piperine moderately impeded *B. sphaericus* (ATCC 14577), *B*. *subtilis* (ATCC 6051), *E*. *coli* (ATCC 25922), *P*. *syringae* (ATCC 13457), and *S*. *typhimurium* (ATCC 23564) and yielded a MIC of 3.9 µg/mL with *S*. *aureus* [47,87]. Piperine (200 µL of the 10 mg/mL/11 mm well) muzzled *S*. *aureus*, *P*. *aeruginosa*, and *C*. *albicans* [87]. Piperine is an antibiotic potentiator that inhibits mycobacterial efflux pumps [50,87]. Piperine very strongly constrained *albicans* [87] and at the concentration of 100 µg/mL suppressed *R*. *solani*, *Fusarium gramineum*, *Alternaria tenuissima*, *Gloeosporium theae-sinensis*, *Phytophthora capsici*, and *Phomopsis adianticola* by 63.1, 53, 66.1, 76.9, 41.8, and 29.3%, respectively [88]. Piperlongumine (200 µL of the 10 mg/mL/11 mm well) from *Piper longum* L. is active against *S*. *aureus* and *P*. *aeruginosa* increased the susceptibility of *S*. *aureus* to rifampicin [87] and very strongly inhibited *C*. *albicans* (MIC: 3.9 µg/mL) [87].

### 10.2. Quinolizidine Alkaloids

From L-lysine are produced quinolizidine alkaloids [89] in the Nymphaeaceae and Fabaceae (Figure 1). 6,6′-Dihydroxythiobinupharidine very strongly suppressed *E*. *faecalis* and *Enterococcus faecium* with the MIC ranging from 2 to 4 µg/mL and MRSA with a MIC of 2 µg/mL [90]. 7-Hydroxylupanineand *N*-butylcytisine from *Sophora flavescens* Aiton (clade Fabids) hindered *S. aureus* with the MIC values of 16 µg/mL [90] and *N*-butylcytisine yielded the MIC of 8 µg/mL with *E. coli* [90].

### 10.3. Phenanthroindolizidine Alkaloids

Plants in the family Lauraceae (clade Magnoliids), Moraceae (clade Fabids, and Asclepiadaceae (clade Lamiids) combine cinnamic acid and ornithine to yield strong broad-spectrum antifungal phenanthroindolizidine alkaloids [91] (Figure 1). One such alkaloid is tylophorinine, which suppressed *C. albicans* (14503), *Candida krusei*, *Candida glabrata*, and *A. fumigatus* with MIC values of 0.6, 0.6, 2.5, and 5 µg/mL, respectively [92]. Tylophorinidine yielded MIC values of 2, 4, 8, and 8 µg/mL with *C. albicans* (14503), *C. krusei*, *C. glabrata*, and *A. fumigatus*, respectively [92] (Table 1). 7-Demethoxytylophorine from *Cynanchum atratum* Bunge was very strongly antifungal with *Penicillium italicum* (MIC/MFC: 1.5/6.2 µg/mL) [25] and *Penicillium digitatum* (MIC/MFC: 1.5/12.5 µg/mL) [92].

### 10.4. Securinega Alkaloids

Plants in the family Phyllanthaceae (clade Fabids) produce Securinega alkaloids (Figure 1) such as viroallosecurinine, which is very active against *P. aeruginosa* and *S. aureus* (MIC: 0.4 μg/mL) [93]. Securinine weakly restrained *P. aeruginosa*, *S. aureus*, and *M. smegmatis* [93]. Allosecurinine and *ent*-seco norsecurinine inhibited the growth of a broad-spectrum of filamentous fungi [94,95].

### 10.5. Miscellaneous

Dihydrodioscorine (0.1% of agar) from *Dioscorea bulbifera* L. inhibited the mycelial growth and spore production of *Sclerotium rolfsii*, *C. lunata*, *F*. *moniliforme*, *Botryodiplodia theobromae*, and *Macrophomina phaseolina* [8]. Pandamarilactone-1 weakly restrained *E*. *coli*, *P*. *aeruginosa*, and *S*. *aureus* [96]. Among the Chenopodiaceae, haloxyline B moderately restrained *M*. *tuberculosis* H37Rv with the MIC of 50 µg/mL [97].

## 11. Quinoline Alkaloids

### 11.1. Simple Quinolines

From tryptophan via anthranilic acid with condensation with malonyl CoA plants in the clade Fabids yielded simple quinolines (Figure 1). 4-Methylquinoline hindered *S*. *aureus* (KCCM 11335) with the MIC/MBC values of 12.2/50 µg/mL [98]. *Lunasia amara* Blanco yielded 4-methoxy-2-phenylquinoline with MIC values of 16 µg/mL against *M*. *tuberculosis* H37Rv [98]. 

In the family Rutaceae (clade Malvids), the condensation of anthranilic acid with malonyl CoA followed by prenylation yielded antibacterial furanoquinolines such as dictamine from *Dictamnus albus* L. hindered *Micrococcus luteus* (TISTR 884) and *B*. *cereus* (TISTR 688) with the MIC values of 26 and 64 µg/mL, respectively [99,100] and was active against *M*. *tuberculosis* H37Rv [101]. γ-Fagarine and robustine are moderate broad-spectrum antibacterial furanoquinolines [100,101]. 

Members of the genus *Gomphandra* Wall. ex Lindl. are produced from the condensation of tryptamine and secologanin yields via strictodamide camptothecin, which is a strong broad-spectrum antifungal pyrrolquinoline alkaloid [102,103].

### 11.2. Benzylisoquinolines

In basal angiosperms (clade Magnoliids, Eudicots), the condensation of dopamine and p-hydroxyphenylacetaldehyde yielded strong broad-spectrum antibacterial and antifungal benzylisoquinoline alkaloids (Figure 1). One such alkaloid is reticuline [104,105]. From *Fumaria indica* Pugsley, fuyuziphine at a concentration of 500 ppm hindered the germination of spores of *Alternaria brassicicola*, *A. solani*, *Alternaria melongenae*, *C. maculans*, *Erysiphe cichoracearum*, and *Helminthosporium pennisetti* by more than 80% [106]. 

### 11.3. Bisbenzylisoquinolines

The radical coupling of benzylisoquinolines gives birth to antibacterial and antifungal bisbenzylisoquinoline alkaloids in plants in the clade Eudicots (Figure 1). For instance, the coupling of *N*-methyl coclaurine yields tetrandine, which is weakly bactericidal for *S*. *aureus* (ATCC 25923) and MRSA (ATCC 33591) [107] and is a bacterial efflux pump inhibitor [90,107].

*Tiliacora triandra* Diels produces tiliacorinine, 2′-nortiliacorinine, and tiliacorine, which achieved MIC values of 6.2, 3.1, and 3.1 µg/mL, respectively, toward *M*. *tuberculosis* (H37Rv) and MIC values ranging from 0.7 to 6.2 µg/mL against several clinical isolates of multidrug-resistant *M*. *tuberculosis* [108]. Tiliacorine inhibited the germination of the conidia of *A. tenuissima* by more than 60% at 100 µg/mL [109].

### 11.4. Aporphines 

The cyclization by oxidative coupling of benzylisoquinolines yields aporphines in basal angiosperms. For instance, in Figure 1, liriodenine [110] inhibited a very broad spectrum of bacteria and phytopathogenic fungi [111] as well as *Histoplasma capsulatum* (MIC: 1.9 μg/mL) [28].

Anonaine developed halos against *B*. *cereus* (ATCC-14.579), *E*. *coli* (ATCC-11.105), *S*. *aureus* (ATCC-6538), and *S*. *epidermidis* (ATCC-12.228) with diameters of 20, 8, 14, and 12 mm, respectively (1 mg/mL, 70 µL/well) [84]. In a subsequent study, anonaine weakly inhibited the growth of *S. mutans* (ATCC 25175) (Lall et al., 2017), *T. rubrum*, and *M. gypseum* [85].

Lysicamine was moderately active with *S*. *epidermidis* (MIC: 50 µg/mL) [112], and very strongly repressed *L*. *monocytogenes*, MSSA, *S*. *pneumoniae*, and *Actinobacillus* sp. with the MIC values of 2.5, 1, 4, and 2.5 µg/mL, respectively, and *K*. *pneumoniae* (ESBL)with the MIC of 20 µg/mL [113]. Lysicamine (10 µg/6 mm disk) developed inhibition zone diameters of 12, 13, and 15.5 mm toward *S*. *epidermidis*, *S. aureus*, and *B. subtilis*, respectively [113], and had meek effects with *Candida dubliniensis* (ATCC 777) [33].

*O*-methylmoschatoline (200 µg/disk) inhibited the growth of *S*. *aureus*, *E*. *coli*, *P*. *aeruginosa*, *S*. *typhi*, and *K*. *pneumoniae* with zone diameters of 20, 12, 12, 20, and 22 mm, respectively [114]. Xylopine hindered *B*. *cereus*, *Micrococcus* sp., and *S*. *aureus* [115] as well as *V*. *cholerae*, *E*. *coli,* and *S*. *dysenteriae* [116]. Artabotrine was very strongly bactericidal against *K*. *pneumoniae* (ESBL) (MIC/MBC: 2.5/2.5 μg/mL) [113]. The azaoxoaporphine sampangine (Log D 2.7 at pH 7.4; molecular weight 232.2 g/mol) was very strongly active with *C*. *albicans* (ATCC 90028), *C*. *glabrata* (ATCC 90030), *C*. *kruseii* (ATCC 6258), *A*. *fumigatus* (ATCC 90906), and *C. neoformans* (ATCC 90113) with MIC values of 3.1, 3.1, 6.2, 6.2, and 0.05 µg/mL, respectively [117] and at 10 µg/disk developed halos with *B. cereus*, *S. aureus*, *E. coli*, and *S. typhi* [118]. Lanuginosine (10 µg/disk) invoked inhibition zone diameters of 12, 14, 10, 14, and 12 mm, with halos with *B. cereus*, *S. aureus*, *E. coli*, *K. pneumoniae*, and *P. aeruginosa*, respectively [118].

Nordicentrine (Log D 3.0 at pH 7.4; molecular weight = 325.3 g/mol) suppressed *M*. *tuberculosis* with a MIC of 12.5 µg/mL [119] as well as *Cladosporium clodosporioides* (6 µg/spot) [120]. Dicentrinone was moderately antimycobacterial [121].

The oxoaporphine thailandine restrained *S*. *pneumoniae*, *S*. *aureus*, and *E*. *faecalis* with MIC values of 30, 30, and 60 µg/mL, respectively, and yielded an IC_50_ value of 6.2 µg/mL with *M*. *tuberculosis* (H_37_Ra) [122]. 

Isoboldine was moderately active with *E*. *coli*, *P*. *aeruginosa*, *P*. *mirabilis*, *K*. *pneumoniae*, *A*. *baumanii*, *S*. *aureus*, *B. subtilis* [104], and *C. albicans* [123]. Bulbocapnine restrained *K*. *pneumoniae* and *A*. *baumanii* with the MIC values of 32, 64, 32, 8, 8, 64, and 128 µg/mL respectively [104].

Roemerine is a moderate inhibitor of MRSA (135), *S*. *aureus* (ATCC25913) *C*. *albicans* (SC 5314), *C*. *glabrata* (8535), *C*. *krusei* (4996), *Candida tropicalis* (8915), *Candida parapsilosis* (90018), and *A*. *fumigatus* (7544), respectively [124] and yielded a MIC value of 10 μg/mL with *C*. *albicans* (ATCC 90028) [125].

Magnoflorine moderately repressed *C*. *albicans* (KCTC7965), *C*. *albicans* (KACC30071), *C*. *parapsilosis* var. *parapsilosis* (KACC45480), *T. rubrum*, and *T. mentagrophytes* [126,127].

### 11.5. Protopines

Benzylisoquinolines are precursors of protopine that restrained *E*. *coli*, *P*. *aeruginosa*, *P*. *mirabilis*, *K*. *pneumoniae*, *A*. *baumanii*, *S*. *aureus,* and *B. subtilis* with the MIC values of 32, 64, 32, 8, 8, 64, and 128 µg/mL, respectively [104] as well as *Streptococcus agalactiae* [128]. Protopine inhibited the growth of *C*. *albicans* with the MIC value of 4 µg/mL [104]. Allocryptopine was weakly active with *S*. *aureus*, *P*. *aeruginosa*, *E*. *coli*, and *S*. *agalactiae* [128].

### 11.6. Protoberberines

The cyclization of benzylisoquinolines yielded very strong antibacterial protoberberines such as in pendulamine A from *Polyalthia longifolia* (Sonn.) Thwaites that yielded the MIC of 2, 0.02, 0.2, 0.02, 2, 2, 0.2, and 0.02 µg/mL against *B. subtilis*, *Corynebacterium hoffmanii*, *S. aureus*, *Micrococcus lysodickycus*, *K. pneumoniae*, *P. aeruginosa*, *S. typhi*, and *S. paratyphi A*, respectively [129]. Pendulamine B restrained *Corynebacterium hoffmanii*, *S. aureus*, *S. faecalis*, *S. viridans*, *M. lysodickycus*, *K. pneumoniae*, *P. aeruginosa*, *S. typhi*, and *S. paratyphi* A with the MIC values of 0.02, 0.2, 2, 0.02, 0.02, 2, 0.2, and 0.2 µg/mL, respectively [129].

### 11.7. Spirobenzylisoquinolines

Spirobenzylisoquinolines are derived from benzylisoquinolines via protoberberine. and include (+)-parfumine, fumarophycine and (+)-fumariline (that moderately restrained *E*. *coli*, *P*. *aeruginosa*, *P*. *mirabilis*, *K*. *pneumoniae*, *A*. *baumanii*, *S*. *aureus,* and *B. subtilis* [104].

### 11.8. Benzophenanthridines 

Plants in the clade Eudicots use reticuline as a precursor of benzophenanthridines such as stylopine or sanguinarine L. that moderately repressed *E*. *coli*, *P*. *aeruginosa*, *P*. *mirabilis*, *K*. *pneumoniae*, *A*. *baumanii*, *S*. *aureus,* and *B. Subtilis* [104]. Dihydrosanguinarine was a weak repressor of *E*. *coli* [128]. Sanguinarine impeded *S*. *mutans* (ATCC 25175) with the MIC value of 32 µg/mL [130] as well as *S*. *aureus*, *P*. *aeruginosa*, *E*. *coli,* and *S*. *agalactiae* with MIC values of 31.3, 250, 62.5, 15.6 µg/mL, respectively [128]. 6-Methoxydihydrosanguinarine (10 µg/disk) developed an inhibition zone diameter of about 17 mm with *S*. *aureus* and MRSA [131]. 8-Hydroxydihydrosanguinarine (molecular weight = 349.3 g/mol) inhibited the growth of clinical strains of MRSA with MIC ranging from 0.4 to 7.8 µg/mL and MBC ranging from 1.9 to 31.2 µg/mL, ESBL strains of *E*. *coli* with MIC ranging from 15.6 to 250 µg/mL and MBC ranging from 62.5 to 500 µg/mL, and *E*. *coli* with MIC of 15.6 µg/mL and MBC of 31.2 µg/mL [109]. 8-Hydroxydihydrosanguinarine was moderately active against *C*. *parapsilosis*, *C*. *tropicalis*, *C*. *krusei*, *C*. *glabrata*, and *C. neoformans* [132].

6-Methoxydihydrosanguinarine is a very strong inhibitor of *E*. *faecalis* and *S*. *aureus* (ATCC 25925) with the MIC/MBC of 5/10, 2.5/5 µg/mL, respectively, and evoked milder potencies with *E*. *coli* (ATCC 25922) [133] and *C*. *albicans* (CMCC 85021) [133]. Norsanguinarine is moderately antibacterial [104]. 

Allocryptopine is the precursor of dihydrochelerythrine that strongly hindered *S*. *epidermidis* (ATCC 12228), *S*. *aureus* (ATCC 6538), *S*. *pyogenes* (ATCC 19615), *B. subtilis* (ATCC 6633), *K*. *pneumoniae* (ATCC 13883), and *E. coli* (ATCC 25922) with MIC/MBC values of 6.2/12.5, 12.5/50, 12.5/50, 25/50, 12.5/25, 25/25 µg/mL, respectively [134]. 8-Hydroxydihydrochelerythrine (molecular weight = 365.4 g/mol) inhibited the growth of 20 strains of MRSA clinical isolated with MIC ranging from 0.9 to 15.6 µg/mL and MBC ranging from 7.8 to 62.5 µg/mL [135] and displayed meeker effects with *E*. *coli* and *K*. *pneumoniae* (ESBL) [109,135]. Dihydrochelerythrine suppressed MRSA (SK1) and *E*. *coli* (TISTR 780) with the MIC values of 8 and 16, and 128 µg/mL [100]. Dihydrochelerythrine is the precursor of chelerythrine, which very strongly hindered *S*. *epidermidis* (ATCC 12228), *S*. *aureus* (ATCC 6538), *S*. *pyogenes* (ATCC 19615), *B. subtilis* (ATCC 6633), *K*. *pneumoniae* (ATCC 13883), and *E*. *coli* (ATCC 25922) with MIC/MBC values of 1.5/12.5, 1.5/3.1, 1.5/6.2, 1.5/50, 1.5/50, and 1.5/25 µg/mL, respectively [128,134]. Chelerythrine was also very strongly antifungal with *C*. *albicans* (ATCC 10231), *S*. *cerevisae* (ATCC 2601), and *C*. *neoformans* (ATCC 28952) with MIC/MBC values of 3.1/3.1, 6.2/6.2, and 3.1/6.2 µg/mL, respectively [134,136]. Corynoline and acetylcorynoline from *Corydalis incisa* (Thunb.) Pers. evoked inhibitory halos with *Cladosporium herbarum* (3 µg/spot) [137]. 

Norchelerythrine inhibited the growth of *M*. *tuberculosis* with the MIC value of 25 µg/mL [138]. Avicine very strongly suppressed *S*. *epidermidis* (ATCC 12228), *S*. *aureus* (ATCC 6538), *S*. *pyogenes* (ATCC 19615), *B*. *subtilis* (ATCC 6633), *K*. *pneumoniae* (ATCC 13883), and *E. coli* (ATCC 25922) with the MIC/MBC values of 3.1/12.5, 1.5/25, 1.5/12.5, 1.5/6.2, and 6.2/12.5 µg/mL, respectively [134]. 8-Acetylnorchelerythrine yielded the MIC values of 1 µg/mL with *S*. *epidermidis*, *E*. *coli*, *E*. *cloacae*, *K*. *pneumoniae*, and *P*. *aeruginosa* [139]. Rhoifoline B moderately inhibited the growth of *S*. *aureus*, *S*. *epidermidis*, *E*. *coli*, *E*. *cloacae*, *K*. *pneumoniae*, *P*. *aeruginosa*, and *S*. *dysenteriae* [139]. Nitidine, from a member of the genus *Zanthoxylum* L. (clade Malvids), weakly inhibited the growth of *M*. *luteus*, *S*. *aureus*, and *M*. *smegmatis* [140].

### 11.9. Protoberberines

Reticuline is precursor of the broad-spectrum antibacterial berberine [29,128,141,142,143,144]. Of note, berberine curbed the growth of *K*. *pneumonia* and *A*. *baumanii* with the MIC value of 8 µg/mL [104] and *Neisseria gonorrhea* with the MIC of 13.5 µg/mL [145]. Berberine decreased the MICs of ampicillin and oxacillin against MRSA and evoked a synergistic effect [146] and impeded the MexAB antibiotic efflux pump in *P*. *aeruginosa* [147]. Berberine suppressed fluconazole-resistant clinical strains of *C. tropicalis*, *C*. *albicans*, *C. parapsilosis*, and *C. neoformans* as well as *C. krusei* (ATCC 6258) and *C. parapsilosis* (ATCC 22019) with the MIC values of 8, 8, 8, 8, 8, 8, 8, 16, 16, 16, 16, 4, and 16 µg/mL, respectively [148] and at a concentration of 500 ppm blocked the germination of spores of *Curvularia lunata*, *Erysiphe cichoracearum*, *F. udum*, and *Penicillium* sp. by more than 80% [106]. Berberine at the concentration of 1.9 µg/mL decreased the MIC of fluconazole from 1.9 to 0.4 µg/mL [149] and inhibited the growth of *M. smegmatis* (ATCC 607) with the MIC value of 25 µg/mL [150].

Berberine is a precursor of jatrorrhizine that curbed strains of *P*. *acnes* with MIC between 5 and 50 µg/mL, [143]. In a subsequent study, jatrorrhizine weakly inhibited *S*. *aureus*, *B*. *subtilis*, *E*. *coli*, and *S*. *dysenteriae* [151] as well as the clinical isolates of *E*. *floccosum*, *T*. *rubrum*, *Trichophyton interdigitale*, *Trichophyton violaceum*, *T*. *mentagrophytes*, *T*. *equinum*, *M*. *canis*, *M*. *gypseum*, *C*. *albicans*, and *C*. *tropicalis* [152]. Palmatine t is moderately antibacterial as well as a sinactine [104] and MexAB antibiotic efflux pump blocker in *P*. *aeruginosa* [147]. 

### 11.10. Phthalides 

Protoberberine alkaloids are precursors of phthalides with moderate antibacterial potencies including adlumidine or bicuculline [104]. Bicuculline at 200 ppm restrained the spore germination of *A. brassicae*, *Curvularia lanata*, and *F. udum* [104,153].

### 11.11. Hasubanans

Within the family Menispermaceae, intramolecular coupling of benzylisoquinolines form hasubanan alkaloids. One such alkaloid is glabradine, isolated from the tubers of *Stephania glabra* (Roxb.) Miers, which suppressed *S*. *aureus* and *S*. *mutans* with the MIC value of 50 µg/mL as well as *M*. *gypseum*, *M*. *canis*, and *T*. *rubrum* with the MIC values of 25, 25, and 50 µg/mL, respectively [154].

### 11.12. Amaryllidaceae Alkaloids

Plants in the family Amaryllidaceae (clade Monocots) condensate protocatechuic aldehyde and tyramine to yield antibacterial Amaryllidaceae alkaloids such as crinamine [9]. Other examples are lycorine and lycoricidine (1 mg/mL/8 mm disk), which developed an inhibition zone of 13 and 12 mm with *E*. *coli*, respectively [106]. Lycorine at the concentration of 100 µg/mL hindered *Alternaria oleracea*, *C*. *gloeosporioides*, *F*. *graminearum*, *Colletotrichum ophiopogonis*, and *Pleospora lycopersici* by 61.9, 57.2, 63.7, 63.2, and 52.6%, respectively [155]. Lycorine yielded the MIC values of 39, 32, 512, 64, and 97.3 µg/mL with *C*. *albicans*, *Candida dubium*, *C*. *glabrata*, *Lodderomyces elongisporus*, and *S*. *cerevisae*, respectively [156]. Narciclasine inhibited the growth of *Corynebacterium fascians* and *C*. *neoformans* [157] and hippeastrine moderately curbed *C*. *albicans* [156]. Tazettine from *Narcissus tazetta* L. weakly restrained *C*. *dubliniensis* and *L*. *elongisporus* [156].

### 11.13. Miscellaneous

#### 11.13.1. Quinolinones

Plants in the clade Malvids yield antibacterial and antifungal quinolinones. Antidesmone at the concentration of 50 µg/mL restrained *Botryosphaeria dothidea*, *Colletotrichum musae*, *Pestalotipsis guepinii*, *Colletotrichum orbiculare*, *Phylophthora nicotianae*, *Pestalotiopsis longiseta*, Carbendazim-sensitive strains of *S*. *sclerotiorum*, and Carbendazim-resistant strains of *S*. *sclerotiorum* by 71.9, 84.6, 86.7, 75.5, 78.8, 83.4, 56.5, and 100%, respectively [158]. Waltherione C at the concentration of 50 µg/mL inhibited *B*. *dothidea*, *Colletotrichum musae*, *Pestalotipsis guepinii*, *Colletotrichum orbiculare*, *Phylophthora nicotianae*, *Pestalotiopsis longiseta*, carbendazim-sensitive strains of *S*. *sclerotiorum*, and carbendazim-resistant strains of *S*. *sclerotiorum* by 60, 43, 71.4, 60.9, 41.6, 3.6, 79.8, and 81.8%, respectively [158].

Members of the genus *Euodia* J.R. Forst. & G. Forst. bring to being series of antibacterial (Gram-positive) long chain quinolinones born of the condensation of anthranilic acid, malonyl-CoA and fatty acyl-CoA (also produced by Gram-negative bacteria). 1-Methyl-2-nonyl-4(1H)-quinolone very strongly repressed *S*. *aureus* (ATCC 25393), *S*. *epidermidis* (ATCC 12228), and *B*. *subtilis* (ATCC 6633) with the MIC values of 4, 4, and 8 µg/mL, respectively [159]. 1-Methyl-2-[(*Z*)-5′-pentadecenyl]-4(1H)-quinolone isolated from the fruits hindered *S*. *aureus* (ATCC 25393), *S*. *epidermidis* (ATCC 12228), and *B*. *subtilis* (ATCC 6633) with the MIC values of 16, 4, and 16 µg/mL, respectively [159]. In a subsequent study, the lipophilic long chain quinolinone alkaloid evocarpine inhibited the growth of MRSA (ATCC 33591) and *S*. *aureus* (ATCC 25923) with the MIC values of 8 and 8 µg/mL, respectively [160] and very strongly suppressed *M*. *fortuitum* (ATCC 6841), *M*. *smegmatis* (ATCC 19429), and *M*. *phlei* (ATCC 19249) with the MIC values of 2, 2, and 2 µg/mL, respectively [161].

#### 11.13.2. Acridanones

In the family Rutaceae (clade Malvids), the condensation of anthranilate with 3 malonyl CoA yielded acridanones such as 1-hydroxy-3,4-dimethoxy-10-methylacridan-9-one [162] or 1-hydroxy-3-methoxy-10-methyl-acridan-9-one that repressed *E*. *coli* [69] (Table 1).

#### 11.13.3. Phenanthrene Alkaloids

Benzylisoquinolines are precursors of antibacterial phenanthrene in basal clades as aristolochic acid, which inhibited the growth of *Moraxella catarrhalis* (GTC 01544) with MIC/MBC values of 25/50 µg/mL [163] as well as *B. subtilis* and *E*. *coli* [164], *P*. *aeruginosa*, *S. faecalis*, *S*. *aureus,* and *Staphylococcus epidermidis* [165]. Another example is aristolactam *N*-(6′-trans-p-coumaroyl)-β-d-glucopuyranoside, which restrained *B. subtilis* and *S*. *lutea* with the MIC values of 43.8 and 175 µg/mL, respectively [166]. These phenanthrene alkaloids bind covalently with DNA [167,168]. 1-*N*-monomethylcarbamate-argentinine-3-*O*-β-d-glucoside at 500 µg/disk hindered MRSA with an inhibition zone diameter of 8 mm [169].

## 12. Pyrrolidines and Imidazole Alkaloids

In the family Piperaceae (clade Magnoliids), the pyrrolidines brachyamide B yielded the IC_50_ of 41.8 µg/mL against a clinical isolate of *C*. *albicans* [170] and suppressed *C*. *neoformans* (ATCC 90 113) with an IC_50_ of 7.1 µg/mL [171]. Other instances are trachyone and isopiperolein B with *S*. *aureus* (MIC of 30 and 36 µM, respectively) [51]. The pyrrolidine alkaloid lactone pandamarilactonine A moderately inhibited the growth of *E*. *coli*, *P*. *aeruginosa*, and *S. aureus* [96]. The pyrrolidine amide *N*-[9-(3,4-Methylenedioxyphenyl)-2E,4E,8E-nonatrienoyl] pyrrolidine was active toward *M*. *tuberculosis* (H37Ra MIC: 25 µg/mL) [170]. 

The imidazole alkaloid allantoin very strongly hindered *B. subtilis* with the MIC value of 4 µg/mL as well as *S*. *aureus*, *E*. *coli*, and *K. pneumoniae* with the MIC value of 8, 8, and 8 µg/mL, respectively [172] (Table 1).

## 13. Diterpene Alkaloids

Plants in the family Ranunculaceae (clade Eudicots) produce a unique type of moderately antifungal diterpene alkaloids. For instance, 8-acetylheterophyllisine, vilmorrianone, and panicutine inhibited the growth of *Allescheria boydii*, *A*. *niger*, *E*. *floccosum*, and *Pleurotus ostreatus* [173] (Table 1). 

## 14. Steroidal Alkaloids

*N*-formylconessimine and conimine suppressed MSSA and MRSA with MIC values of 32 and 128 μg/mL, respectively, and conimine increased the sensitivity of MSSA to vancomycin [174].

## 15. Concluding Remarks

Weinstein and Albersheim (1983) presented evidence that phytoalexins, especially flavonoids from angiosperms, act as nonspecific membrane antimicrobials that alter the structural integrity of the cytoplasmic membrane, thereby causing the membrane to be a less efficient matrix for membrane-dependent processes [175]. They also argue that phytoalexins with non-specific antimicrobial targets targeting the cytoplasm and proteins also makes it difficult for bacteria or fungi to develop resistance. Furthermore, phytoalexins are often toxic to herbivorous predators as well as repellent and therefrom exhibit low therapeutic indices. In the case of alkaloids, none have been known act on specific antibacterial or antifungal targets [176]. It is for this reason that antibiotic or antifungal alkaloids for systemic use in humans working at micromolar plasmatic concentrations have not been found yet. Research efforts, however, need to continue with the determination of the selectivity indices. Alkaloids from Asian angiosperms represent yet another mind-blowing source of original chemical frameworks that can be used for the hemisynthesis of clinical antibiotics, antimycobacterial agents, and antifungal drugs as well as efflux pump inhibitors of clinical value. 8-Acetylnorchelerythrine, cryptolepine, 8-hydroxydihydrochelerythrine, 6-methoxydihydrosanguinarine, 2′-nortiliacorinine, pendulamine A and B, rhetsisine, sampangine, tiliacorine, tryptanthrin, tylophorinine, vallesamine, and viroallosecurinine with a MIC ≤1 µg/mL are first line candidates.

## Figures and Tables

**Table 1:** The chemical structures of antibacterial and/or antifungal alkaloids and their botanical origin.

CLASS Compound	Chemical Structure	Genus, Species	Family
ACRIDANONES			
1-Hydroxy-3,4-dimethoxy-10-methylacridan-9-one	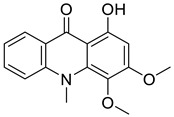	*Limonia acidissima* L.	Rutaceae
1-Hydroxy-3-methoxy-10-methylacridan-9-one	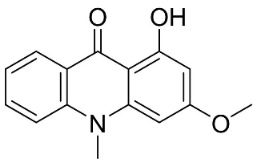	*Limonia acidissima* L.	Rutaceae
AMARYLLIDACEAE ALKALOIDS			
Crinamine	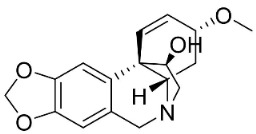	*Crinum asiaticum* L.	Amaryllidaceae
Lycoricidine	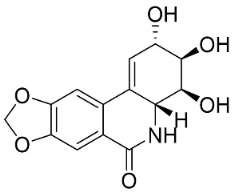	*Lycoris radiata* (L’Hér.) Herb.	Amaryllidaceae
Lycorine	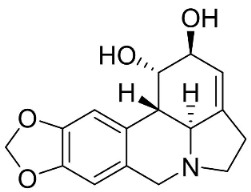	*Lycoris radiata* (L’Hér.) Herb.	Amaryllidaceae
Narciclasine	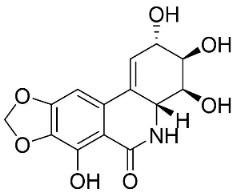	*Lycoris radiata* (L’Hér.) Herb.	Amaryllidaceae
Tazettine	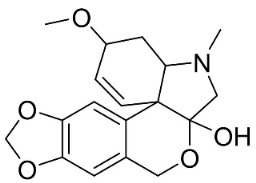	*Narcissus tazetta* L.	Amaryllidaceae
APORPHINES			
Anonaine	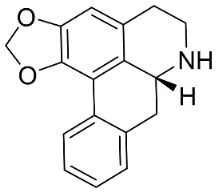	*Michelia alba* DC.	Magnolicaceae
Artabotrine	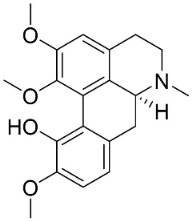	*Artabotrys suaveolens* (Bl.) Bl.	Annonaceae
Bulbocapnine	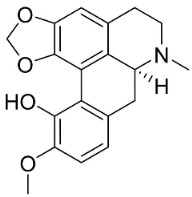	*Corydalis bulbosa* DC.	Fumariaceae
Dicentrinone	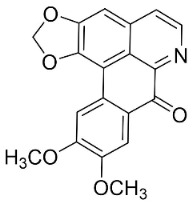	*Phoebe lanceolata* (Nees) Nees	Lauraceae
Isoboldine	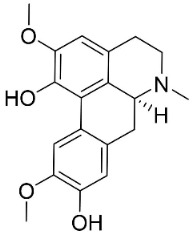	*Corydalis bulbosa* DC.	Fumariaceae
Lanuginosine	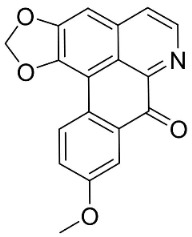	*Eupomatia laurina* R. Br.	Eupomatiaceae
Liriodenine	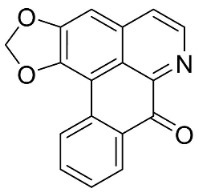	*Cananga odorata* Hook. F. & Thomson	Annonaceae
Lysicamine	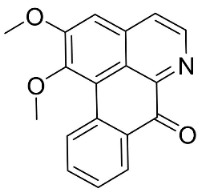	*Phoebe grandis* (Nees) Merr.	Lauraceae
Magnoflorine	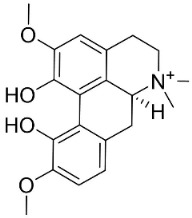	*Mahonia bealei* (Fortune) Carrière	Papaveraceae
Nordicentrine	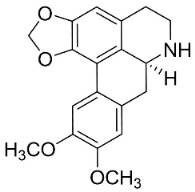	*Phoebe lanceolata* (Nees) Nees	Lauraceae
*O*-Methylmoschatoline	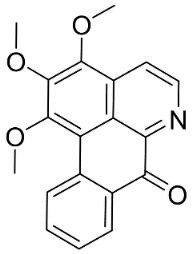	*Cananga odorata* (Lam.) Hook. F. & Thomson	Annonaceae
Roemerine	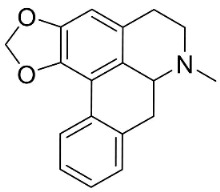	*Papaver rhoeas* L.	Papaveraceae
Sampangine	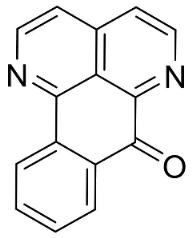	*Eupomatia laurina* R. Br.	Eupomatiaceae
Thailandine	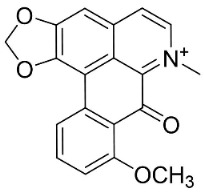	*Stephania venosa* (Bl.) Spreng	Menispermaceae
Xylopine	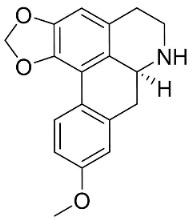	*Artabotrys suaveolens* (Bl.) Bl.	Annonaceae
BENZOPHENANTHRIDINES			
8-Acetylnorchelerythrine	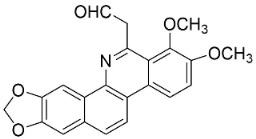	*Toddalia asiatica* (L.) Lam.	Rutaceae
Avicine	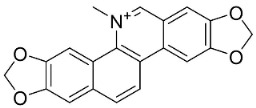	*Toddalia asiatica* (L.) Lam.	Rutaceae
Chelerythrine	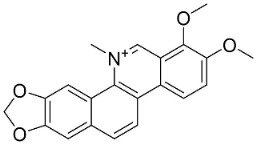	*Macleaya cordata* (Willd.) R. Br.	Papaveraceae
Corynoline	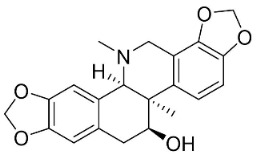	*Corydalis incisa* (Thunb.) Pers.	Fumariaceae
Dihydrochelerythrine	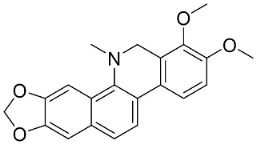	*Macleaya cordata* (Willd.) R. Br.	Papaveraceae
Dihydrosanguinarine	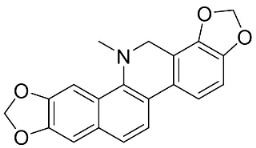	*Macleaya cordata* (Willd.) R. Br	Papaveraceae
8-Hydroxydihydrochelerythrine	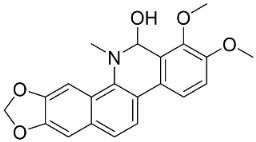	*Chelidonium majus* L.	Fumariaceae
8-Hydroxydihydrosanguinarine	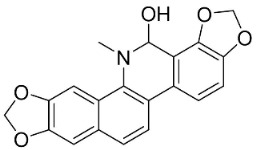	*Chelidonium majus* L.	Fumariaceae
6-Methoxydihydrosanguinarine	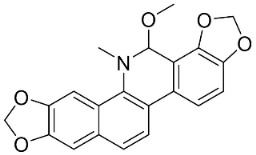	*Chelidonium japonicum* Thunb	Fumariaceae
Nitidine	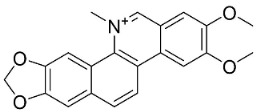	*Zanthoxylum* L.	Rutaceae
Norchelerythrine	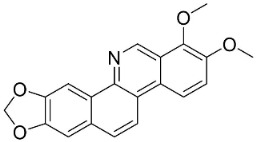	*Toddalia asiatica* (L.) Lam.	Rutaceae
Norsanguinarine	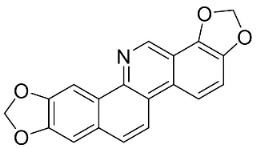	*Fumaria indica* Pugsley	Fumariaceae
Rhoifoline B	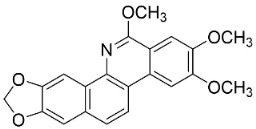	*Toddalia asiatica* (L.) Lam.	Rutaceae
Sanguinarine	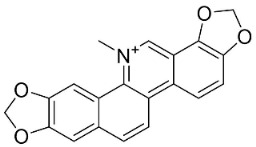	*Fumaria officinalis* L.	Fumariaceae
Stylopine	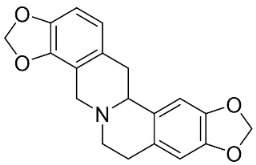	*Fumaria officinalis* L.	Fumariaceae
CARBAZOLES			
3,3′-[Oxybis(methylene)]bis(9-methoxy-9*H*-carbazole)	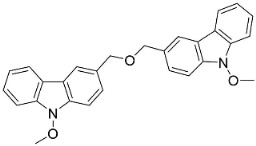	*Murraya koenigii* (L.) Spreng.	Rutaceae
Clausamine A	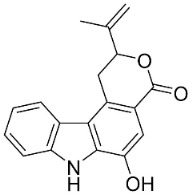	*Clausena harmandiana* (Pierre) Guillaumin	Rutaceae
Clausamine B	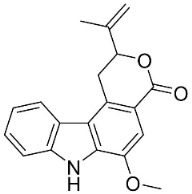	*Clausena harmandiana* (Pierre) Guillaumin	Rutaceae
Clausine F	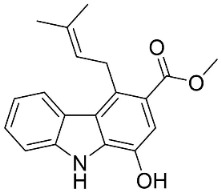	*Clausena harmandiana* (Pierre) Guillaumin	Rutaceae
Clauszoline N	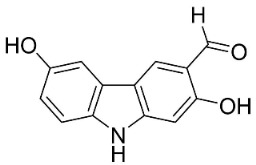	*Clausena harmandiana* (Pierre) Guillaumin	Rutaceae
3-Formylcarbazole	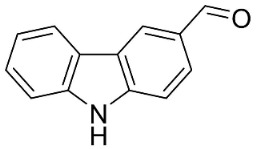	*Clausena mexicana* Burm.f.	Rutaceae
3-Formyl-1-methoxycarbazole	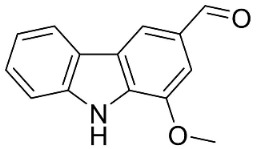	*Murraya koenigii* (L.) Spreng.	Rutaceae
Girinimbine	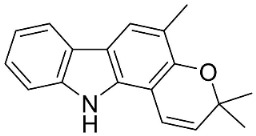	*Murraya koenigii* (L.) Spreng.	Rutaceae
Glycozolidol	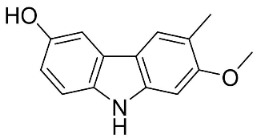	*Glycosmis pentaphylla* (Retz.) DC.	Rutaceae
Harmane	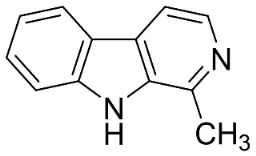	*Murraya mexicana* (L.) Jack	Rutaceae
Koenimbine	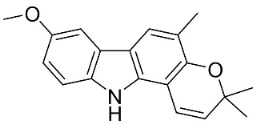	*Murraya koenigii* (L.) Spreng.	Rutaceae
Koenigine	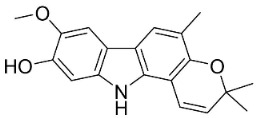	*Murraya koenigii* (L.) Spreng.	Rutaceae
Lansine	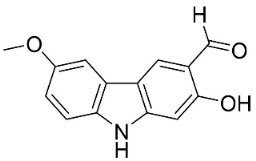	*Micromelum pubescens* Bl.	Rutaceae
Murrayamine J	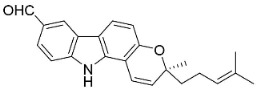	*Murraya mexicana* (L.) Jack	Rutaceae
BENZYLISOQUINOLINES			
Reticuline	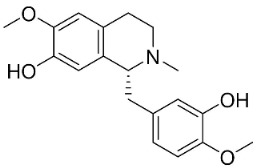	*Annona squamosa* L.	Annonaceae
Fuyuziphine	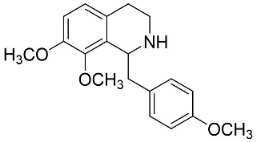	*Fumaria indica* Pugsley	Fumariaceae
BISBENZYLISOQUINOLINES			
2′-Nortiliacorinine	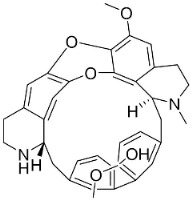	*Tiliacora triandra* Diels	Menispermaceae
Tetrandrine	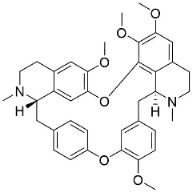	*Cyclea barbata* Miers	Menispermaceae
Tiliacorine	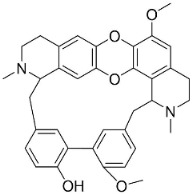	*Tiliacora triandra* Diels	Menispermaceae
Tiliacorinine	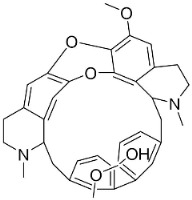	*Tiliacora triandra* Diels	Menispermaceae
DITERPENE ALKALOIDS			
8-Acetylheterophyllisine	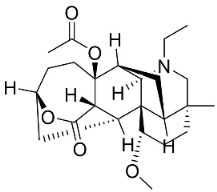	*Delphinium denudatum* Wall. Ex Hook. F. & Thomson yields	Ranunculaceae
Panicutine	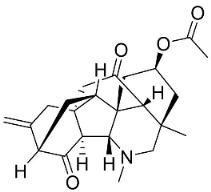	*Delphinium denudatum* Wall. Ex Hook. F. & Thomson yields	Ranunculaceae
Vilmorrianone	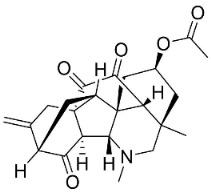	*Delphinium denudatum* Wall. Ex Hook. F. & Thomson yields	Ranunculaceae
HASUBANANS			
Glabradine	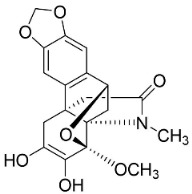	*Stephania glabra* (Roxb.) Miers	Menispermaceae
IMIDAZOLES			
Allantoin	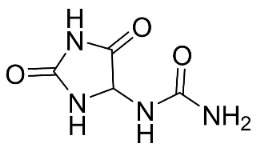	*Tournefortia sarmentosa* Lam	Borraginaceae
INDOLOQUINAZOLINES			
Dehydroevodiamine	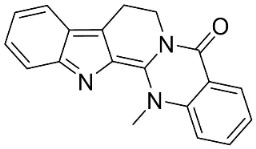	*Euodia rutaecarpa* Benth	*Rutaceae*
Evodiamine	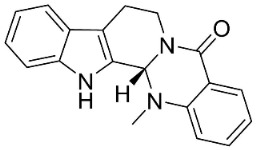	*Euodia rutaecarpa* Benth	*Rutaceae*
Tryptanthrin	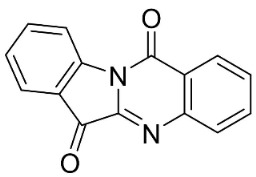	*Indigofera tinctoria* L.	*Fabaceae*
INDOLOQUINOLINES			
Cryptolepine	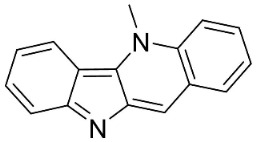	*Cryptolepis sanguinolenta* (Lindl.) Schltr.	Apocynaceae
MONOTERPENE INDOLE ALKALOIDS			
Alstoniascholarine A	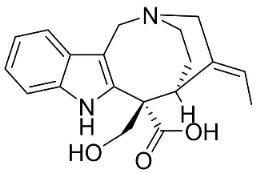	*Alstonia scholaris* (L.) R.Br.	*Apocynaceae*
Alstoniascholarine E	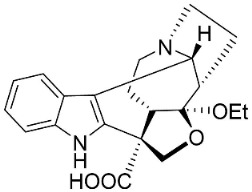	*Alstonia scholaris* (L.) R.Br.	*Apocynaceae*
Alstoniascholarine J	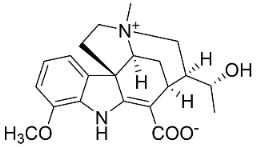	*Alstonia scholaris* (L.) R.Br.	*Apocynaceae*
Cadambine	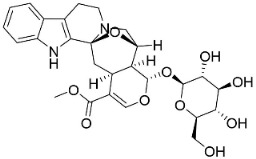	*Neolamarckia cadamba* (Roxb.) Bosser	*Rubiaceae*
Ibogaine	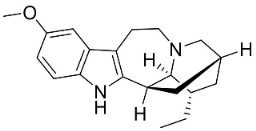	*Ervatamia mexicana* (L.) Burkill	*Apocynaceae*
3-Oxocoronaridine	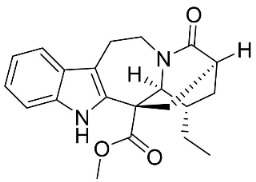	*Ervatamia mexicana* (L.) Burkill	*Apocynaceae*
5-Oxocoronaridine	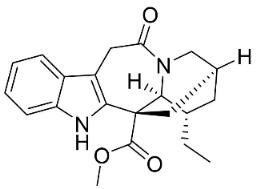	*Ervatamia mexicana* (L.) Burkill	*Apocynaceae*
Strictosidine	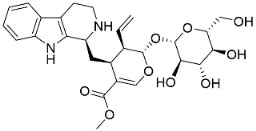	*Guettarda* speciosa L.	*Rubiaceae*
Vallesamine	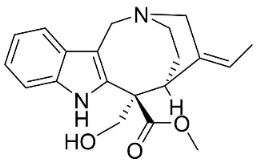	*Ervatamia mexicana* (L.) Burkill	*Apocynaceae*
Voacamine	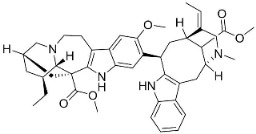	*Ervatamia mexicana* (L.) Burkill	*Apocynaceae*
Voacangine	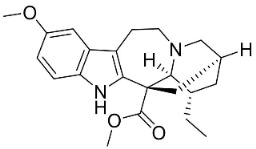	*Ervatamia exicana* (L.) Burkill	*Apocynaceae*
Vobasine	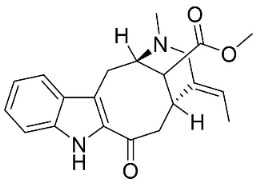	*Ervatamia mexicana* (L.) Burkill	*Apocynaceae*
Tubotaiwine	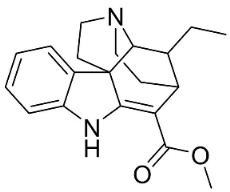	*Alstonia scholaris* (L.) R.Br.	*Apocynaceae*
PHENANTHRENE ALKALOIDS			
Aristolochic acid	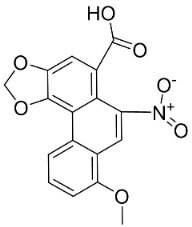	*Aristolochia* L.	*Aristolochiaceae*
Aristolactam *N*-(6′-trans-p-coumaroyl)-β-d-glucopyranoside	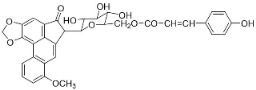	*Aristolochia* L.	*Aristolochiaceae*
1-*N*-monomethylcarbamate-argentinine-3-*O*-β-d-glucoside	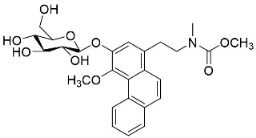	*Stephania succifera* H.S. Lo & Y. Tsoong	*Meninspermaceae*
PHENANTHROINDOLIZIDINE ALKALOIDS			
7-Demethoxytylophorine	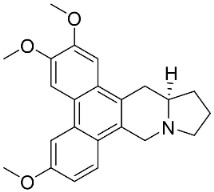	*Cynanchum atratum* Bunge	*Asclepiadaceae*
Tylophorinine	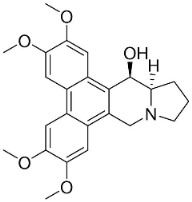	*Tylophora indica* (Burm.f.) Merr.	*Asclepiadaceae*
Tylophorinidine	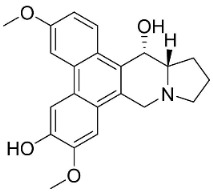	*Tylophora indica* (Burm.f.) Merr	*Asclepiadaceae*
PIPERINE ALKALOIDS			
Piperine	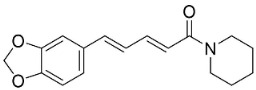	*Piper nigrum* L.	Piperaceae
Piperlongumine	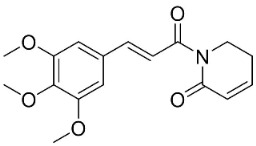	*Piper longum* L.	Piperaceae
PHTHALIDES			
Adlumidine	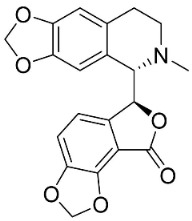	*Fumaria officinalis* L. t	Fumariaceae
Bicuculline	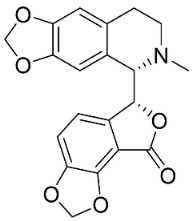	*Corydalis bulbosa* DC.	Fumariaceae
PROTOBERBERINES			
Berberine	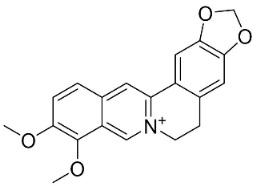	*Coptis chinensis Franch*.	Ranunculaceae
Jatrorrhizine	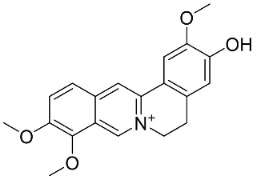	*Coptis chinensis Franch*.	
Palmatine	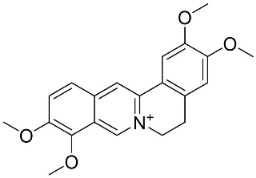	*Corydalis exicana* (Thunb.) Pers.	Fumariaceae
Sinactine	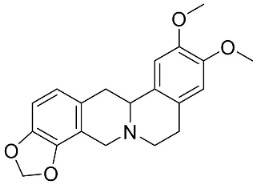	*Fumaria officinalis* L.	Fumariaceae
PYRROLIDINES			
Brachyamide B	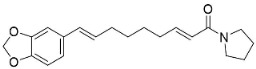	*Piper nigrum* L.	Piperaceae
Isopiperolein B	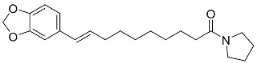	*Piper nigrum* L.	Piperaceae
*N*-[9-(3,4-Methylenedioxyphenyl)-2E,4E,8E-nonatrienoyl]pyrrolidine	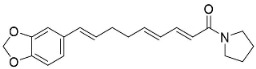	*Piper nigrum* L.	Piperaceae
Pandamarilactonine A	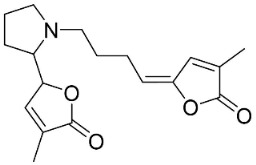	*Pandanus odorus* Ridl.	Pandanaceae
Trachyone	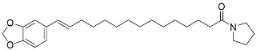	*Piper nigrum* L.	Piperaceae
QUINOLINONES			
Antidesmone	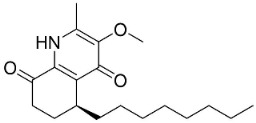	*Waltheria indica* L.	Malvaceae
Evocarpine	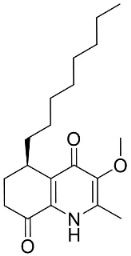	*Euodia rutaecarpa* Benth	Rutaceae
1-Methyl-2-nonyl-4(1H)-quinolone	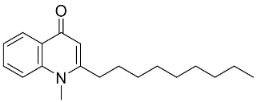	*Euodia rutaecarpa* Benth	Rutaceae
1-Methyl-2-[(*Z*)-5′-pentadecenyl]-4(1H)-quinolone	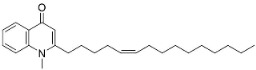	*Euodia rutaecarpa* Benth	Rutaceae
Waltherione C	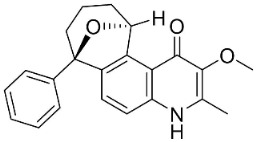	*Waltheria indica* L.	Malvaceae
QUINOLIZIDINE ALKALOIDS			
6,6′-Dihydroxythiobinupharidine	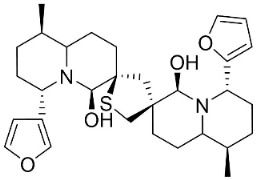	*Nuphar japonica* DC.	Nymphaeaceae
7-Hydroxylupanine	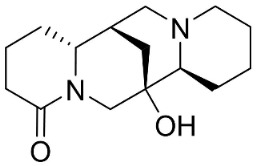	*Sophora flavescens* Aiton	Fabaceae
*N*-Butylcytisine	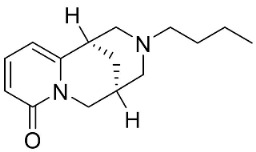	*Sophora flavescens* Aiton	Fabaceae
SECURINEGA ALKALOIDS			
Securinine	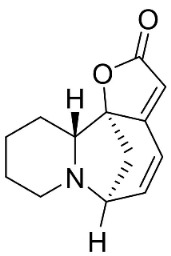	*Flueggea virosa* (Roxb. Ex Willd.) Royle	Phyllanthaceae
Viroallosecurinine	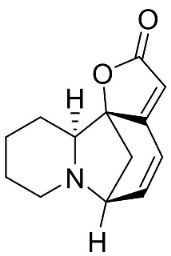	*Flueggea virosa* (Roxb. Ex Willd.) Royle	Phyllanthaceae
MISCELLANEOUS PIPERINE ALKALOIDS			
Dihydrodioscorine	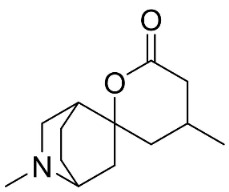	*Dioscorea bulbifera* L.	Dioscoreaceae
Haloxyline B	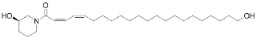	*Haloxylon salicornicum* (Moq.) Bunge ex Boiss.	Chenopodiaceae
Pandamarilactone-1	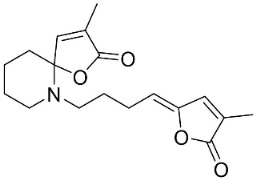	*Pandanus odorus* Ridl.	Pandanaceae
SIMPLE QUINOLINE ALKALOIDS			
Camptothecin	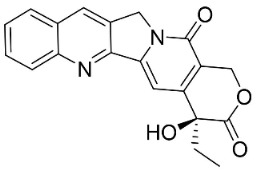	*Gomphandra* Wall. Ex Lindl.	Icacinaceae
Dictamine	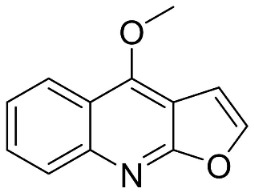	*Dictamnus albus* L.	Rutaceae
γ-Fagarine	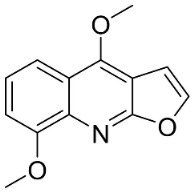	*Dictamnus albus* L.	Rutaceae
4-Methoxy-2-phenylquinoline	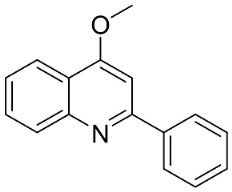	*Lunasia amara* Blanco	Rutaceae
4-Methylquinoline	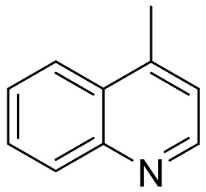	*Citrullus colocynthis* (L.) Schrad.	Cucurbitaceae
Robustine	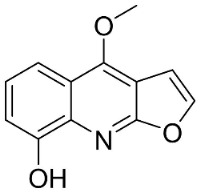	*Dictamnus albus* L.	Rutaceae
PROTOBERBERINES			
Pendulamine A	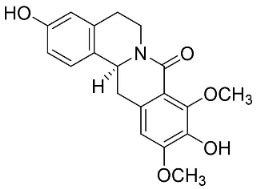	*Polyalthia longifolia* (Sonn.)	Annonaceae
Pendulamine B	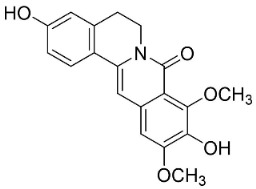	*Polyalthia longifolia* (Sonn.)	Annonaceae
PROTOPINES			
Allocryptopine	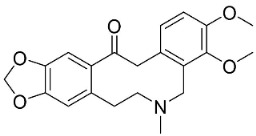	*Macleaya cordata* (Willd.) R. Br	Papaveraceae
Protopine	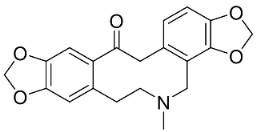	*Argemone mexicana* L.	
SPIROBENZYLISOQUINOLINES			
(+)-Fumariline	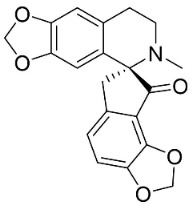	*Fumaria officinalis* L.	Fumariaceae
Fumarophycine	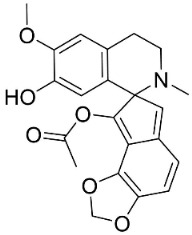	*Fumaria officinalis* L. yields	Fumariaceae
(+)-Parfumine	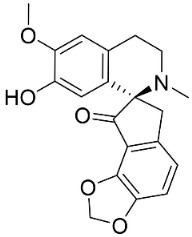	*Fumaria indica* Pugsley	Fumariaceae
STEROIDAL ALKALOIDS			
Conimine	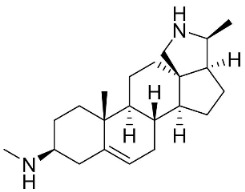	*Holarrhena pubescens* Wall. Ex G. Don	Apocynaceae
*N*-Formylconessimine	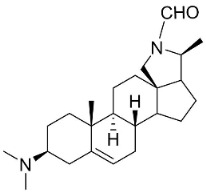	*Holarrhena pubescens* Wall. Ex G. Don	Apocynaceae

## Supplementary Materials

The following supporting information can be downloaded at: https://www.mdpi.com/article/10.3390/antibiotics11091146/s1, Table S1: Medicinal Plants of Asia and the Pacific yielding antibacterial and/or antifungal alkaloids; Table S2: Antibacterial and/or antifungal alkaloid *in vitro* and from Asian Angiosperms with MIC ≤ 5 µg/mL.
