# Supplementary material for: Antibacterial and Antifungal Alkaloids from Asian Angiosperms: Distribution, Mechanisms of Action, Structure-Activity, and Clinical Potentials
Source: Antibiotics (Basel). 2022 Aug 24;11(9):1146. doi: 10.3390/antibiotics11091146 (PMC9495154; doi:10.3390/antibiotics11091146)
Supplement: Supplementary file 1 [file antibiotics-11-01146-s001.zip › Table S1.pdf]

Table S1. Medicinal Plants of Asia and the Pacific yielding antibacterial and/or antifungal alkaloids

Clade	Family	Genus, species	Local name	Use	Country
Protomagnoliids	Nymphaeaceae	*Nuphar japonica* DC.	Senkotsu	Wounds	Japan
Magnoliids	Aristolochiaceae	*Asarum sieboldii* Miq.	Jok do ri	Fever	Korea
		Aristolochia L.			
	Annonaceae	*Annona squamosa* L.	Ates	Fever	the Philippines
		Artabotrys suaveolens (Bl.) Bl.	Akar chenana	Cholera	Indonesia
		Cananga odorata Hook. F. and Thom		Boils	Solomon Islands
		Goniothalamus laoticus (Finet & Gagnep.) Bân			
		Polyalthia longifolia (Sonn.) Thwaites	Debdaru	Skin infection	Bangladesh
	Eupomatiaceae	*Eupomatia laurina* R. Br.			
	Lauraceae	*Phoebe grandis* (Nees) Merr.			
		Phoebe lanceolata (Nees) Nees	Changpichla	Leucorrhoea	Bangladeh
	Magnoliaceae	*Magnolia grandiflora* L.			
		Michelia alba DC.	Cempaka puteh	Post-partum	Malaysia
	Piperaceae	*Piper longum* L.	Pipul	Tuberculosis	Bangladesh
		Piper nigrum L.f,	Hu jiao	Cholera	China
Monocots	Amaryllidaceae	*Lycoris radiata* (L'Hér.) Herb.	Shi suan	Abscesses	China
		Narcissus tazetta L.	Shui xian	Abscesses	China
	Dioscoreaceae	*Dioscorea bulbifera* L.	Man soen	AIDS	Thailand
	Pandanaceae	*Pandanus odorus* Ridl.	Shanlha	Syphilis	Bangladesh
Eudicots	Berberidaceae	*Mahonia bealei* (Fortune) Carrière	Kuo ye shi da gong lao	Tuberculosis	China
	Fumariaceae	*Chelidonium japonicum* Thunb.			
		Chelidonium majus L.	Sarılık out	Jaundice	Turkey
		Corydalis bulbosa DC.		Boils	China
		Corydalis incisa (Thunb.) Pers.	Ke ye zi jin	Abscesses	China
		Corydalis racemosa (Thunb.) Pers.	Xiao hua huang jin	Abcesses	China
		Fumaria indica Pugsley	Pitpapra	Fever	India
		Fumaria officinalis L.	Shaahtara	Fever	India
	Menispermaceae	*Cyclea barbata* Miers	Cincau hijau	Typhoid	Indonesia
		Stephania glabra (Roxb.) Miers	Muchi lota	Mycosis	Bangladesh
		Stephania venosa (Blume) Spreng			
		Stephania succifera H.S. Lo & Y. Tsoong	Xiao ye di bu rong	Detoxification	China
		Tiliacora triandra Diels	Choi nang	Fever	Thailand
	Papaveraceae	*Argemone mexicana* L.	Druju	Ulcers	Indonesia
		Macleaya cordata (Willd.) R. Br.	Bo luo hui	Ringworm	China
		Papaver rhoeas L.	Kopekyagi	Cough	Turkey
	Ranunculaceae	*Coptis chinensis* Franch.	Huang lian	Antiseptic	China
		Delphinium denudatum Wall. ex Hook. f. & Thomson	Nirbasi	Ulcers	India
Fabids	Brassicaceae	*Capsella bursa-pastoris* (L.) Medik	Phol	Dysentery	Pakistan
	Cucurbitaceae	*Citrullus colocynthis* (L.) Schrad.	Tumba	Leprosy	Pakistan
	Fabaceae	*Indigofera tinctoria* L.	Nilayamari	Bronchitis	India

		Sophora flavescens Aiton			
	Fagaceae	*Quercus dentata* Thunb.	Hu shu	Sores	China
	Phyllanthaceae	*Flueggea virosa* (Roxb. ex Willd.) Royle	Botolan	Boils	the Philippines
		Phyllanthus amarus Schumach. & Thonn.			
	Rhamnaceae	*Ziziphus mauritiana* Lam.	Putrea	Gingivitis	Vietnam

Table 2. Continuation

Clade	Family	Genus, species	Local name	Use	Country
Malvids	Amaranthaceae	*Achyranthes ferruginea* Roxb.		Boils	Bangladesh
	Chenopodiaceae	*Haloxylon salicornicum* (Moq.) Bunge ex Boiss.	Lana	Wounds	Pakistan
	Malvaceae	*Melochia corchorifolia* L.		Dysentery	India
	Simaroubaceae	*Brucea javanica* (L.) Merr.	Kom roi	Fever	Laos
	Nyctaginaceae	*Mirabilis jalapa* L.	Gul e Abas	Syphilis	Pakistan
	Nitrariaceae	*Peganum harmala* L.		Fever	Pakistan
	Rutaceae	*Clausena excavata* Burm.f.	Daw-hke	Leprosy	Daw-hke
		Clausena harmandiana (Pierre) Guillaumin			
		Dictamnus albus L.		Fever	India
		Euodia rutaecarpa Benth			
		Lunasia amara Blanco			
		Glycosmis pentaphylla (Retz.) DC.	Athishadla	Boils	Bangladesh
		Micromelum pubescens Bl.		Tuberculosis	India
		Murraya koenigii (L.) Spreng.	Kari patta	Fever	Bangladesh
		Murraya paniculata (L.) Jack			
		Toddalia asiatica (L.) Lam.	Dahanbaz-dahan	Jaundice	Iran
		Zanthoxylum L.			
	Simaroubaceae	*Brucea javanica* (L.) Merr.	Dahun belur	Ringworm	Indonesia
	Sterculiaceae	*Waltheria indica* L.		Syphilis	the Philippines
Lamiids	Acanthaceae	*Strobilanthes cusia* (Nees) Kuntze			
	Apocynaceae	*Alstonia scholaris* (L.) R.Br.	Watsil	Fever	Papua New Guinea
		Ervatamia divaricata (L.) Burkill	Tagar	Eye infection	Bangladesh
		Rauvolfia serpentina (L.) Benth. ex Kurz	Bomma yaza	Gonorrhoea	Myanmar
		Holarrhena pubescens Wall. ex G. Don	Dangkyam	Boils	Bangladesh
	Asclepiadaceae	*Cynanchum atratum* Bunge		Wounds	Korea
		Tylophora indica (Burm.f.) Merr.	Untomul	Dysentery	Bngladesh
	Boraginaceae	*Tournefortia sarmentosa* Lam	Salakapo kapu	Fever	Papua
	Gentianaceae	*Gentiana macrophylla* Pallas	Qin jiao	Tuberculosis	China
	Icacinaceae	*Gomphandra* Wall. ex Lindl.			
	Rubiaceae	*Borreria verticillata* (L.) G. Mey.	Nathaisoor	Gonorrhoea	India
		Guettarda speciosa L.	Hinma	Wounds	India

	Neolamarckia cadamba (Roxb.) Bosser	Kadam	Wounds	Nepal
Asteraceae	*Spilanthes paniculata* Wall. ex DC		Dysentery	India
