# Supplementary material for: Antibacterial and Antifungal Alkaloids from Asian Angiosperms: Distribution, Mechanisms of Action, Structure-Activity, and Clinical Potentials
Source: Antibiotics (Basel). 2022 Aug 24;11(9):1146. doi: 10.3390/antibiotics11091146 (PMC9495154; doi:10.3390/antibiotics11091146)
Supplement: Supplementary file 1 [file antibiotics-11-01146-s001.zip › Table S2.pdf]

Table S2. Antibacterial and/or antifungal alkaloid *in vitro* and from the medicinal plants of Asia and the Pacific with MIC ≤ 5 μg/mL

Microorganism	Alkaloid	Class	LM	MM	HM	HP	AP	LP	PSA	P
**Gram-positive cocci**	Avicine	Benzophenantridine		367.7		+			40.8	+
	8-Acetyl norchelerythrine*	Benzophenantridine		375			+			+
	Chelerythrine	Benzophenantridine		348.4		+			40.8	+
	Clausamine B	Carbazole		307.3			4.5		51.3	+
	Cryptolepine	Indoloquinoline		232.2		1.4			17.8	+
	6,6′-Dihydroxythiobinupharidine	Quinolizidine			526.7		3.5		98.5	
	8-Hydroxydihydrochelerythrine*	Benzophenantridine		365.4		+			60.4	+
	8-Hydroxydihydrosanguinarine	Benzophenantridine		349.3		+			60.4	+
	Lysicamine*	Aporphine		291.3			3.6		48.4	+
	6-Methoxydihydrosanguinarine*	Benzophenantridine		363.4					48.4	+
	1-Methyl-2-nonyl-4(1H)-quinolone	Quinolone		285.5				6.4	20.3	
	Methyl-2-[(Z)-5′-pentadecenyl]-4(1H)-quinolone	Quinolone						+		
	Norchelerythrine	Benzophenantridine		333.3		+			49.8	+
	Pendulamine A*	Protoberberine		341			+			+
	Pendulamine B	Protoberberine		339						+
	Piperine	Piperine		285.3			2.5		38.8	
	Tryptanthrin*	Indoloquinazoline			248.2			2.4	49.7	+
	Vallesamine	Corynanthe indole			340.4			+	65.6	+
	Viroallosecurinine*	Securinega			217.2			+	29.5	
**Gram-positive bacilli**	Avicine	Benzophenantridine		367.7		+			40.8	+
	Lysicamine	Aporphine		291.3			3.6		48.4	+
	Norchelerythrine	Benzophenantridine		333.3		+			49.8	+
	Pendulamine A*	Protoberberine		341			+			
	Pendulamine B*	Protoberberine		339			+			+
**Gram-negative bacilli**	8-Acetyl norchelerythrine*	Benzophenantridine		375			+			+
	Artabotrine	Aporphine		341.1			3.1		51.2	+
	Chelerythrine	Benzophenantridine		348.4		+			40.8	+
	Dehydroevodiamine	Indoloquinazoline			301.3				35.9	+
	Harman	Carbazole		182.2			+		28.7	+
	Lysicamine	Aporphine		291.3			3.6		48.4	+
	Norchelerythrine	Benzophenantridine		333.3		+			49.8	+

	Pendulamine A*	Protoberberine		341			+			
	Pendulamine B*	Protoberberine		339			+			+
	Rhetsisine*	β-carboline		319.3			2.9		62.5	+
	Vallesamine*	Corynanthe indole			340.4			+	65.6	+
	Viroallosecurinine*	Securinega		217.2			+		29.5	

Table 2. Continuation

Microorganism	Alkaloid	Class	LM	MM	HM	HP	AP	LP	PSA	P
**Mycobacteria**	Evocarpine	Quinolinones		339.5				5.9	20.3	
	2′-Nortiliacorinine*	Bisbenzylisoquinoline				562.7		5.6	72.4	
	Tiliacorine*	Bisbenzylisoquinoline				576.6		5.7	63.6	
**Yeasts**	Chelerythrine	Benzophenantridine		348.4		+			40.8	+
	Liriodenine	Aporphine		275.0			3.3		48	+
	Piperlongumine	Piperine		317.3			1.6			65
	Sampangine*	Aporphine		232.2			2.7		43	+
	Tylophorinine *	Phenanthroindolizidine		379.4			3.5		51	+
	Tylophorinidine	Phenanthroindolizidine		365.4			2.7		62	+
	Tryptanthrin	Indoloquinazoline		248.2			2.4		49.7	+
**Filamentous fungi**	Cryptolepine*	Indoloquinoline		232.2		1.4			17.8	+
	7-Demethoxytylophorine	Phenanthroindolizidine		363.4			4.3		30.9	+
	Sampangine*	Aporphine		232.2			2.7		43	+
	Tylophorinine	Phenanthroindolizidine		379.4			3.5		51	+

*: MIC ≤ 1 μg/mL; LM: Low molecular mass (g/mol); MM: Medium molecular mass (g/mol) ; HM: High molecular mass (g/mol); HP: Hydrophilic; AP: Amphiphilic; LP: Lipophilic; PNA: Polar surface area (Å^2); P: Planar
